# Transcriptomics reveals metabolic division of labor and reproduction-metabolism decoupling in *Saccharomyces cerevisiae* biofilms

**DOI:** 10.1016/j.isci.2026.117407

**Published:** 2026-09-09

**Authors:** Caice Liang, Jing Ruan, Biao Yang, Wei Liu, Qingguo Liu, Chaowei Zhou, Junqiang Shan, Shuqi Shi, Hui Han, Wenjun Sun, Yong Chen

**Affiliations:** 1 National Engineering Research Center for Biotechnology, College of Biotechnology and Pharmaceutical Engineering, Nanjing Tech University, Nanjing, China; 2 State Key Laboratory of Materials-Oriented Chemical Engineering, College of Biotechnology and Pharmaceutical Engineering, Nanjing Tech University, Nanjing 211816, China

**Keywords:** biofilm structure heterogeneity, biofilms, fermentation process and gene expressions, *Saccharomyces cerevisiae*, transcriptome analysis

## Abstract

Biofilm-based manufacturing offers superior robustness, yet the spatial logic governing its metabolic resilience remains a “black box,” hindering rational strain engineering. Here, we present a spatiotemporal transcriptomic map of *Saccharomyces cerevisiae* biofilms under industrial conditions. We identify a fundamental “reproduction-metabolism decoupling” strategy: the biofilm functions as a self-organizing system where outer cells act as a “metabolic shield”—activating retrograde signaling to endure stress and maximize glycolysis—while inner cells serve as a “protected nursery” for rapid proliferation. This spatial division of labor explains biofilm stability and provides a genetic blueprint for spatially programming microbial factories for next-generation biomanufacturing.

## Highlights

• BIF exhibits superior fermentation performance over FCF
• Yeast biofilms employ a spatially distinct reproduction-metabolism decoupling
• The outer layer forms a metabolic shield while the inner layer drive cell self-renewal
• The middle layer mainly upregulates *FLO* genes to support biofilm structural stability

## Graphical abstract

**Figure:**
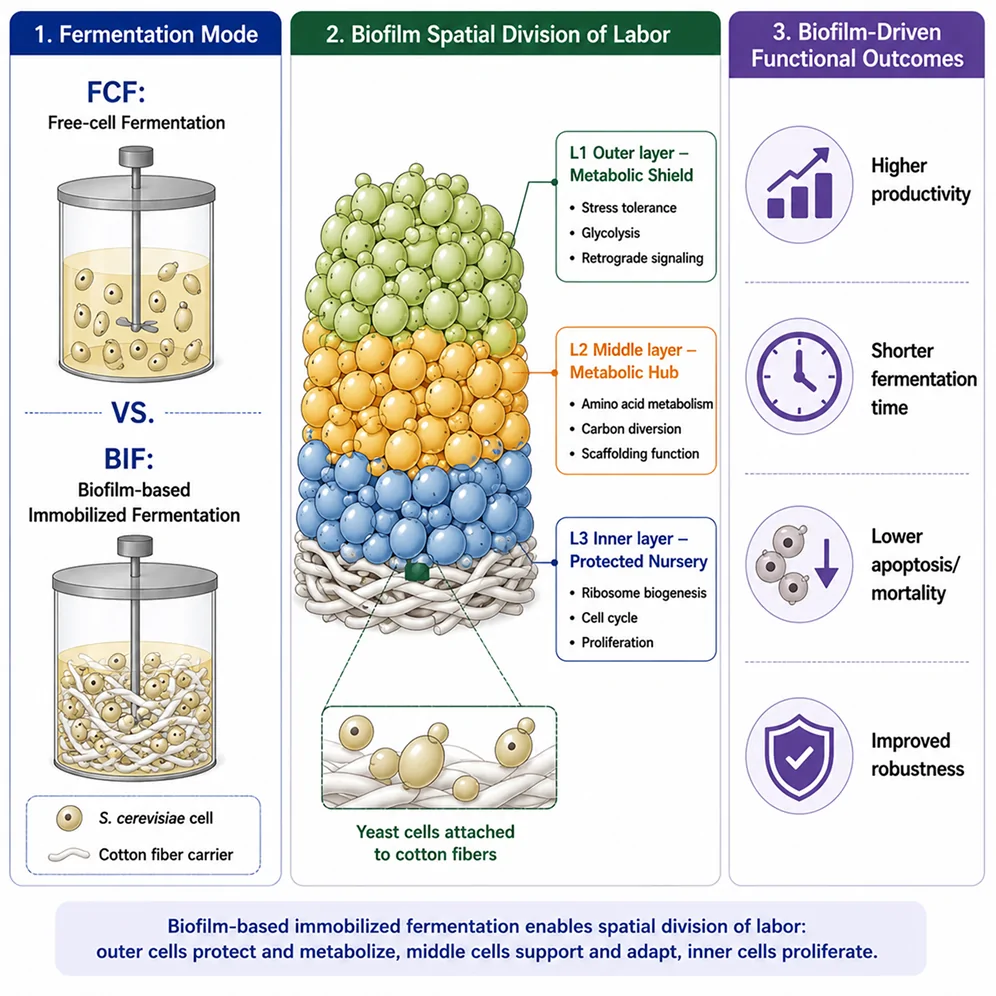

## Teaser

Microbiofilms; Microbiology; Transcriptomics

## Introduction

Biofilm-based fermentation has emerged as a transformative biotechnology in industrial microbiology, exploiting the natural tendency of microorganisms to form surface-adherent communities. Originally defined by Costerton in 1995 as “matrix-enclosed bacterial populations adherent to each other and/or to surfaces or interfaces,”^1^^,^^2^ the concept of biofilms has since evolved to encompass a broad range of biological and industrial applications.^3^^,^^4^^,^^5^^,^^6^^,^^7^^,^^8^ The intrinsic properties of biofilms—including self-immobilization, environmental resilience, and enhanced metabolic activity—position biofilm-based immobilized fermentation (BIF) as a promising alternative to conventional free-**c**ell **f**ermentation (FCF) for industrial ethanol production.^1^^,^^3^^,^^9^^,^^10^^,^^11^

Comparative studies across laboratory and industrial scales consistently demonstrate BIF’s superiority over FCF in key performance metrics. BIF systems achieve higher product titers, yields, productivities, and purities across diverse microbial platforms.^12^^,^^13^^,^^14^ Remarkable improvements include *Escherichia coli* (*E. coli*) biofilms enhancing L-threonine production by 53.44%,^15^ *Clostridium beijerinckii* increasing acetone-butanol-ethanol productivity 50-fold,^16^^,^^17^ and *Saccharomyces cerevisiae* (*S. cerevisiae*) reducing fermentation time consumption by two-thirds.^18^ Furthermore, BIF enables efficient semi-continuous processing with improved product purity, as demonstrated in L-valine production.^14^ Industrial validation through a 320-ton pilot-scale bioreactor confirmed these advantages, showing significant improvements in ethanol titer (12.46% vs. 12.01% v/v) and yield (83.72% vs. 80.52%) compared to FCF.^19^

While synthetic biology has mastered metabolic pathway design in homogeneous planktonic cultures, spatial engineering of microbial communities remains a frontier. Current empirical “black box” approaches fail to exploit the natural 3D metabolic stratification of biofilms. Unlocking this spatial logic is essential to transition from stochastic screening to the rational design of heterogeneous biocatalysts (biofilms). In FCF, genetically identical cell populations respond uniformly to temporal changes in bulk environmental conditions, such as glucose depletion and ethanol accumulation.^20^ Conversely, BIF orchestrates a complex three-dimensional architecture characterized by steep nutrient gradients, differential metabolite accumulation, and heterogeneous cell-cell signaling across distinct biofilm subpopulations.^21^ This spatial heterogeneity creates functionally specialized microenvironments that enable metabolic division of labor and enhanced stress tolerance. Previous investigations have suggested that colony biofilms exhibit position-dependent gene expression patterns linked to metabolic partitioning and stress adaptation,^22^^,^^23^^,^^24^ highlighting the critical importance of understanding spatiotemporal dynamics in biofilm function. However, most of these insights are derived from static colony models on agar plates, and the specific spatial metabolic stratification mechanisms of fungal biofilms under the dynamic, submerged conditions of industrial fermentation remain largely unexplored. This knowledge gap significantly limits our ability to rationally design and optimize biofilm-based fermentation systems for enhanced productivity and robustness.

This study addresses this critical limitation by employing comprehensive transcriptome profiling to systematically resolve the temporal evolution and spatial heterogeneity of gene expression in *S. cerevisiae* biofilms during ethanol fermentation. We provide the systematic analysis of genetic reprogramming across biofilm subpopulations in an industrially relevant context, offering unprecedented molecular insights into biofilm functional organization. These findings establish a fundamental understanding of spatiotemporal gene regulation in biofilm fermentation systems, providing a rational foundation for optimizing biofilm-immobilized fermentation technologies.

## Results

All major abbreviations and operational metrics mentioned in the following results are outlined in Table 1 and further detailed in the STAR Methods.

**Table 1:** Summary of sample nomenclature and experimental groups

Abbreviation	Sample description	Source
FCF	**f**ree-**c**ell **f**ermentation	–
BIF	**b**iofilm-based **i**mmobilized **f**ermentation	–
FP1	the **p**lanktonic **c**ells of FCF at a glucose concentration of 75%	FCF
FP2	the planktonic cells of FCF at a glucose concentration of a half	FCF
FP3	the planktonic cells of FCF at a glucose concentration of 25%	FCF
FP4	the planktonic cells of FCF at the end of fermentation phase	FCF
BP1	the **B**IF’s **p**lanktonic cells at 1 h at batch 8	planktonic cells in BIF
BP2	the BIF’s planktonic cells at 2 h at batch 8	planktonic cells in BIF
BP3	the BIF’s planktonic cells at 3 h at batch 8	planktonic cells in BIF
BP4	the BIF’s planktonic cells at 4 h at batch 8	planktonic cells in BIF
BP5	the BIF’s planktonic cells at 5 h at batch 8	planktonic cells in BIF
B2I1	the biofilm cells obtained after the first vortex oscillation stage (60 W, 15 s) of the carrier at 2 h at batch 8	biofilm cells
B4I1	the biofilm cells obtained after the first vortex oscillation stage (60 W, 15 s) of the carrier at 4 h at batch 8	biofilm cells
B5I1	the biofilm cells obtained after the first vortex oscillation stage (60 W, 15 s) of the carrier at 5 h at batch 8	biofilm cells
B4I2	the biofilm cells obtained after the second vortex oscillation stage (60 W, 15 s) of the carrier at 4 h at batch 8	biofilm cells
B4I3	the biofilm cells obtained after the third vortex oscillation stage (60 W, 15 s) of the carrier at 4 h at batch 8	biofilm cells
B4I4	the biofilm cells obtained after the fourth vortex oscillation stage (60 W, 15 s) of the carrier at 4 h at batch 8	biofilm cells
L1	the first layer of the biofilm cells (the biofilm cells obtained after the first vortex oscillation stage (60 W, 15 s) of the carrier at batch 8: B2I1, B4I1, and B5I1)	biofilm cells
L2	the second layer of the biofilm cells (the biofilm cells obtained after the second vortex oscillation stage (60 W, 15 s) of the carrier at batch 8: B4I2)	biofilm cells
L3	the third layer of biofilm cells (the biofilm cells obtained after the third and the fourth vortex oscillation stage (60 W, 15 s) of the carrier at batch 8: B4I3 and B4I4)	biofilm cells
FPCs	**F**CF’s **p**lanktonic **c**ells: FP1, FP2, FP3, and FP4	–
BPCs	**B**IF’s **p**lanktonic **c**ells: BP1, BP2, BP3, BP4, and BP5	–
BCs	the **b**iofilm **c**ells: L1, L2, and L3	–
SVG1	**s**ignificant **v**aried **g**enes between planktonic cells and L1 cells at 2 h (B2I1 vs. BP2)	–
SVG2	significant varied genes between planktonic cells and L1 cells at 4 h (B4I1 vs. BP4)	–
SVG3	significant varied genes between planktonic cells and L1 cells at 5 h (B5I1 vs. BP5)	–

### BIF outperformed on ethanol production than FCF

**Figure 1:**
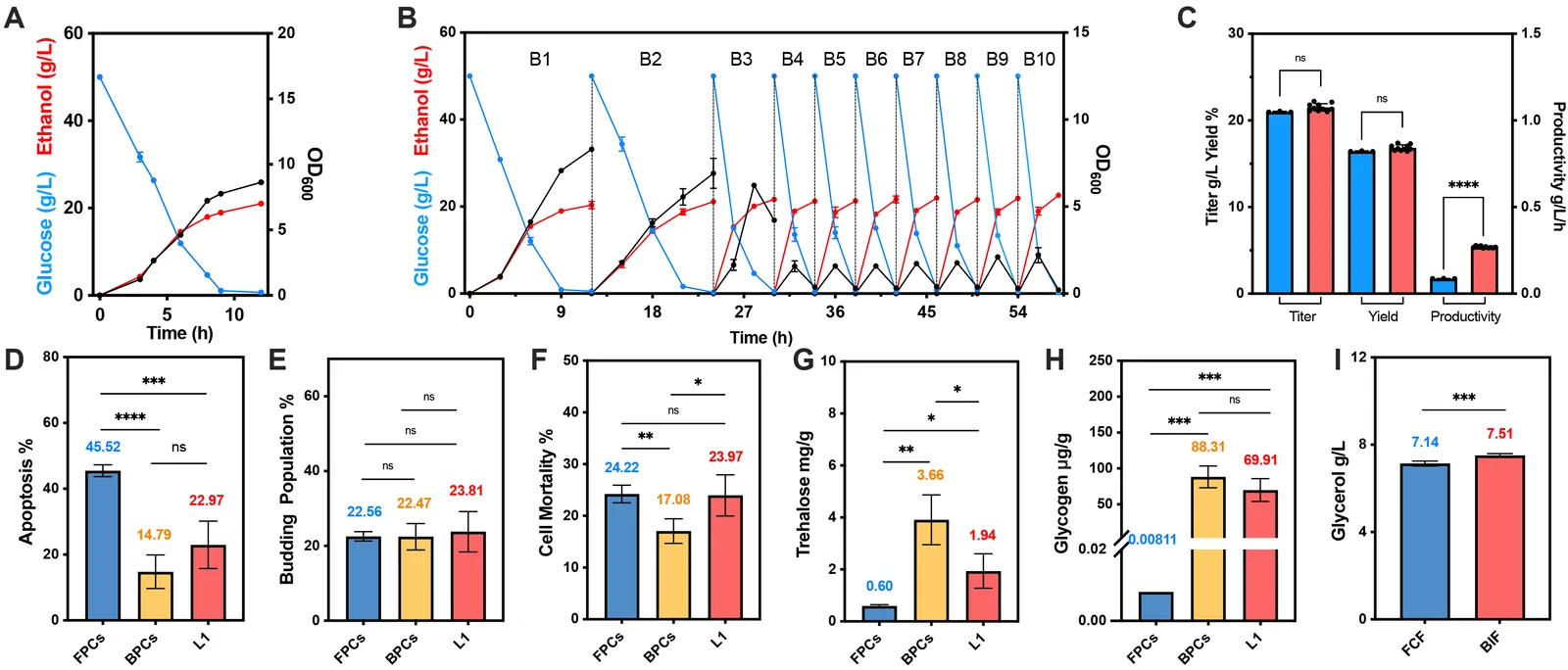
The evaluations of BIF and FCF The fermentation kinetic curves of FCF (A) and BIF (B), and the fermentation abilities of FCF (the blue bars) and BIF (the red bars) (C); cell states, including apoptosis (D), bud populations (E), and cell mortality (F); and other carbon substrates, including trehalose (G), glycogen (H), and glycerol (I). BIF, biofilm-based immobilized fermentation; FCF, free-cell fermentation; FPCs, FCF’s planktonic cells including FP1, FP2, FP3, and FP4; BPCs, BIF’s planktonic cells including BP1, BP2, BP3, BP4, and BP5; L1, the first layer of the biofilm cells (the biofilm cells obtained after the first vortex oscillation stage (60 W, 15 s) of the carrier at batch 8 including B2I1, B4I1, and B5I1). Data are represented as mean ± SEM; ns *p* > 0.05, ∗*p* < 0.05, ∗∗*p* < 0.01, ∗∗∗*p* < 0.001, ∗∗∗∗*p* < 0.0001 by one-way ANOVA (*n* = 3 biological replicates per group).

Biofilm-based biotechnologies have advanced rapidly due to their superior fermentation performance. Our previous study demonstrated that BIF achieved higher titer, yield, and productivity compared to FCF.^19^ FCF required 12 h to produce 21.01 g/L ethanol by consuming 50 g/L glucose (Figure 1A), whereas BIF fermentation time decreased progressively with successive batches, ultimately stabilizing at 4 h (Figure 1B). Throughout the BIF process, each batch achieved marginally higher titers than FCF, except for the initial batch. Overall, BIF exhibited modest improvements in titer and yield but substantial enhancement in productivity (Figure 1C). Cellular viability analysis revealed that FCF displayed significantly elevated apoptosis levels compared to BIF (Figure 1D). Both fermentation systems exhibited similar budding population dynamics (Figure 1E). While overall cell mortality rates were comparable between FCF and BIF, planktonic cells in BIF demonstrated approximately 30% lower mortality than FCF (Figure 1F). At the end of the fermentation process, in BIF, BIF planktonic cells (BPCs) and L1 cells accumulated higher concentrations of trehalose and glycogen relative to FCF from batch 3 to 10 (Figures 1G and 1H). However, glycerol accumulation showed minimal variation, with BIF producing only 0.37 g/L more than FCF (Figure 1I).

### Biofilms maintain transcriptomic stability unlike the “panic response for survival” of free cells in FCF

**Figure 2:**
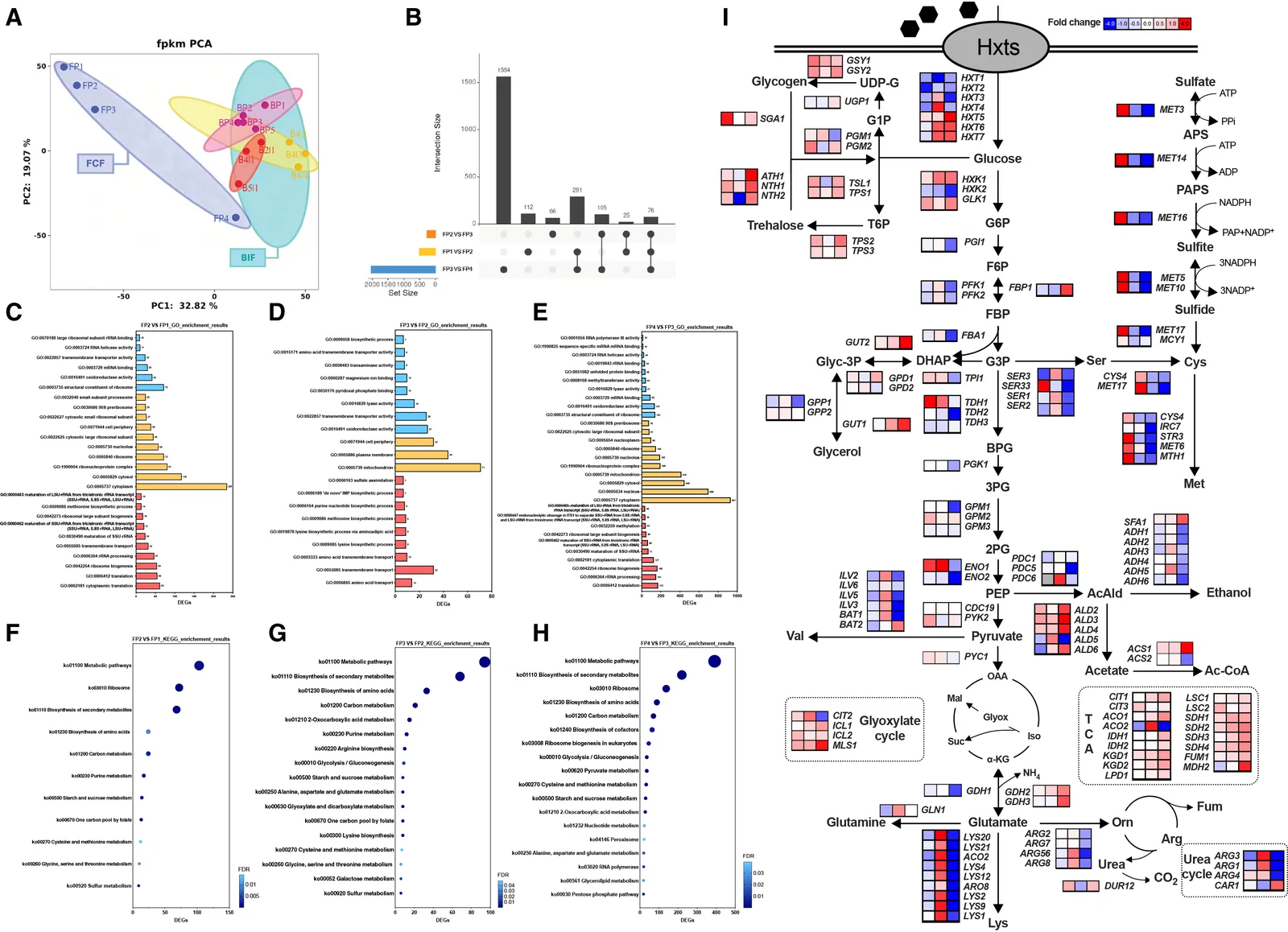
Transcriptome analysis of FPCs in temporal dimension (A) Principal component analysis (PCA) of samples collected from FCF and BIF; (B) overview of DEGs in three groups (FP2 vs. FP1, FP3 vs. FP2, and FP4 vs. FP3); GO (the blue bar: biological progress; the yellow bar: cellular component; the red bar: molecular function) and KEGG enrichment analysis in FP2 vs. FP1 (C and F), FP3 vs. FP2 (D and G), and FP4 vs. FP3 (E and H); and (I) differences in gene expressions related to carbon metabolism (glycolysis, glycogen biosynthesis, trehalose biosynthesis, pyruvate metabolism, TCA, glyoxylate cycle) and nitrogen metabolism (cysteine and methionine biosynthesis, glutamate biosynthesis, lysine biosynthesis, and arginine biosynthesis). The sample names from left to right are FP2 vs. FP1, FP3 vs. FP2, and FP4 vs. FP3. BIF, biofilm-based immobilized fermentation; FCF, free-cell fermentation; FP1, FP2, FP3, and FP4, the planktonic cells of FCF at a glucose concentration of 75%, 50%, 25%, and less than 1 g/L, respectively; BP1, BP2, BP3, BP4, and BP5, the BIF’s planktonic cells at 1, 2, 3, 4, and 5 h, respectively, at batch 8; B2I1, B4I1, and B5I1, the biofilm cells obtained after the first vortex oscillation stage (60 W, 15 s) of the carrier at 2, 4, and 5 h, respectively, at batch 8; B4I2, B4I3, and B4I4, the biofilm cells obtained after the second, third, and forth vortex oscillation stage (60 W, 15 s) of the carrier at 4 h at batch 8. FPCs, FCF’s planktonic cells including FP1, FP2, FP3, and FP4; HXTs, hexose transporters; G6P, glucose-6-phosphate; F6P, fructose-6-phosphate; FBP, fructose-1,6-bisphosphate; G3P, glyceraldehyde 3-phosphate; BPG, 1,3-bisphosphoglycerate; 3 PG, 3-phosphoglycerate; 2 PG, 2-phosphoglycerate; PEP, phosphoenolpyruvate; G1P, glucose-1-phosphate; UDP-G, UDP glucose; T6P, trehalose-6-phosphate; DHAP, dihydroxyacetone phosphate; Glyc-3P, glycerone-3P; Ser, serine; Cys, cysteine; Met, methionine; APS, adenylyl sulfate; PAPS, 3-phosphoadenylyl sulfate; AcAld, acetaldehyde; Ac-CoA, acetyl-CoA; Val, valine; OAA, oxaloacetate; Iso, isocitrate; α-KG, α-ketoglutarate; Suc, succinate; Mal, malate; Lys, lysine; Orn, ornithine; Arg, arginine; Fum, fumarate.

Throughout the manuscript, all differential expression analyses are presented as “experimental group vs. control group,” terms such as “upregulated” or “downregulated” refer to the status of the first group relative to the second excluding specific explanations. Four temporal samples were collected during FCF to investigate transcriptional changes throughout the fermentation process (FP1, FP2, FP3, and FP4). Principal component analysis (PCA) revealed distinct clustering patterns among the four samples, indicating substantial transcriptional differences across fermentation phases (Figure 2A). A total of 2,239 differentially expressed genes (DEGs) were identified throughout the FCF process (Figure 2B), with detailed statistical analysis presented in Table S1. The temporal analysis revealed upregulation of 272, 213, and 842 DEGs and downregulation of 233, 59, and 1,194 DEGs across sequential fermentation phases, respectively.

Gene Ontology (GO) enrichment analysis demonstrated that DEGs were predominantly enriched in cellular component (CC) categories (Figures 2C–2E; Table S2). During the FP2 vs. FP1 comparisons (Figure 2C), DEGs in FP2 were upregulated in protein biosynthesis processes, including ribosome biogenesis (GO:0042254), translation (GO:0006412), and rRNA processing (GO:0006364), supporting rapid cellular proliferation.^25^ As glucose consumption progressed, cells shifted toward transmembrane transport (GO:0055085), amino acid transmembrane transport (GO:0006865), and amino acid biosynthesis pathways (GO:0009085, GO:0019878, GO:0009086, and GO:0000103) (Figure 2D). Upon glucose depletion, DEGs were predominantly enriched in translation (GO:0006412) and ribosome biogenesis (GO:0042254) processes (Figure 2E). Kyoto Encyclopedia of Genes and Genomes (KEGG) pathway enrichment analysis identified key metabolic transitions during FCF, with results categorized into four functional groups: protein-related, carbon-related, amino acid-related, and other pathways (Table S3). During the initial phase (FP2 vs. FP1, Figure 2F), genes of FP2 in ribosome pathways (ko03010) were significantly downregulated (Table S2), indicating reduced protein biosynthesis requirements and resource reallocation toward ethanol production.^26^^,^^27^ Concurrently, DEGs in starch and sucrose metabolism (ko00500) were upregulated (Figure 2G; Table S2). Sulfur metabolism (ko00920) activation led to cysteine accumulation, while methionine accumulation corresponded with upregulation of *CYS4*, *STR3*, *MET6*, and *MTH1* (Figure 2I). Notably, *ADE4* downregulation conserved resources for purine metabolism, thereby decelerating cell growth.^28^ As fermentation progressed (FP3 vs. FP2, Figure 2G), starch and sucrose metabolism (ko00500) was further activated. Upregulation of DEGs in glycolysis/gluconeogenesis supported pyruvate accumulation, activating alanine, aspartate, and glutamate metabolism (ko00250), arginine biosynthesis (ko00220), and lysine biosynthesis (ko00300), while inactivating sulfur metabolism (ko00920) and cysteine and methionine metabolism (ko00270). Upon glucose depletion (Figure 2H, FP4 vs. FP3), ribosome-related pathways (ko03010 ribosome and ko03008 ribosome biogenesis in eukaryotes) were silenced, arresting cell cycle progression. Under these nutrient-depleted conditions, starch and sucrose metabolism (ko00500) remained active to maintain glucose homeostasis, while glycolysis/gluconeogenesis (ko00010) and amino acid metabolisms (ko00270 Cysteine and methionine metabolism, ko00250 Alanine, aspirate, and glutamate metabolism, and ko00300 Biosynthesis of amino acids) were suppressed.

**Figure 3:**
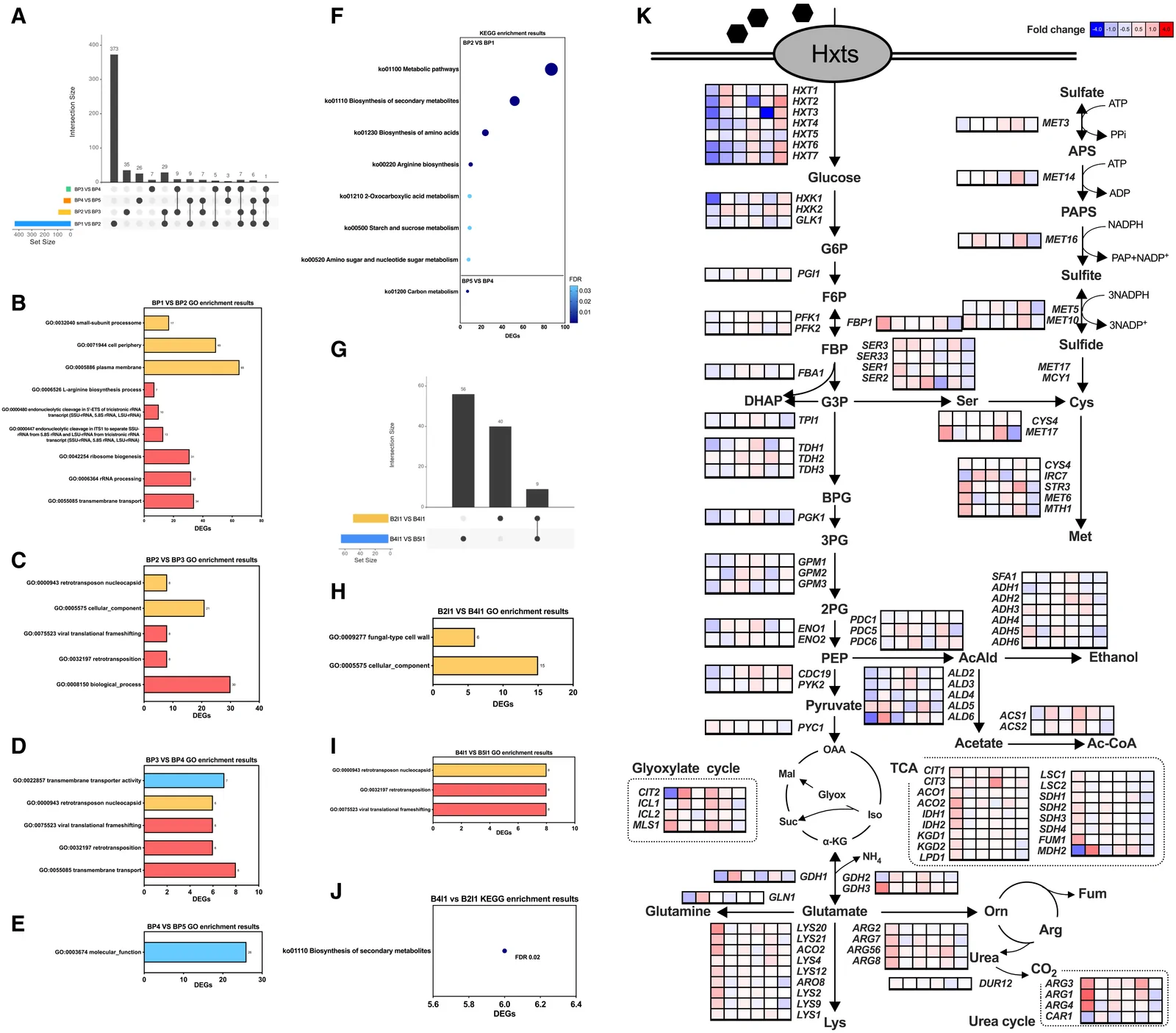
Transcriptome analysis of planktonic cells in biofilm-based immobilized fermentation and L1 cells in temporal dimension (A) Overview of DEGs between BPCs (BP2 vs. BP1, BP3 vs. BP2, BP4 vs. BP3); GO (the blue bar: biological progress; the yellow bar: cellular component; the red bar: molecular function) and KEGG enrichment analysis in BP2 vs. BP1 (B and F), BP3 vs. BP2 (C), BP4 vs. BP3 (D), BP5 vs. BP4 (E and G); (H) overview of DEGs in between L1 cells (B4I1 vs. B2I1 and B5I1 vs. B4I1); GO (I and J) and KEGG (K) enrichment analysis in B4I1 vs. B2I1 (I) and B5I1 vs. B4I1 (J); and difference in gene expressions related to carbon metabolism (glycolysis, glycogen biosynthesis, trehalose biosynthesis, pyruvate metabolism, TCA, glyoxylate cycle) and nitrogen metabolism (cysteine and methionine biosynthesis, glutamate biosynthesis, lysine biosynthesis, and arginine biosynthesis). The sample names from left to right are BP2 vs. BP1, BP3 vs. BP2, BP4 vs. BP3, BP5 vs. BP4, B4I1 vs. B2I1, and B5I1 vs. B4I1. BP1, BP2, BP3, BP4, and BP5, the BIF’s planktonic cells at 1, 2, 3, 4, and 5 h, respectively, at batch 8; B2I1, B4I1, and B5I1, the biofilm cells obtained after the first vortex oscillation stage (60 W, 15 s) of the carrier at 2, 4, and 5 h, respectively, at batch 8; BPCS, BIF’s planktonic cells including BP1, BP2, BP3, BP4, and BP5; HXTs, hexose transporters; G6P, glucose-6-phosphate; F6P, fructose-6-phosphate; FBP, fructose-1,6-bisphosphate; G3P, glyceraldehyde 3-phosphate; BPG, 1,3-bisphosphoglycerate; 3 PG, 3-phosphoglycerate; 2 PG, 2-phosphoglycerate; PEP, phosphoenolpyruvate; DHAP, dihydroxyacetone phosphate; Ser, serine; Cys, cysteine; Met, methionine; APS, adenylyl sulfate; PAPS, 3-phosphoadenylyl sulfate; AcAld, acetaldehyde; Ac-CoA, acetyl-CoA; OAA, oxaloacetate; Iso, isocitrate; α-KG, α-ketoglutarate; Suc, succinate; Mal, malate; Lys, lysine; Orn, ornithine; Arg, arginine; Fum, fumarate.

Five temporal samples of planktonic cells were collected during BIF (BP1, BP2, BP3, BP4, and BP5). PCA revealed tighter clustering of BPCs compared to FCF’s planktonic cells (FPCs), indicating greater transcriptomic stability in planktonic cells of BIF (Figure 2A). Only 517 DEGs were identified throughout the BIF process (Figure 3A), with temporal upregulation of 244, 82, 13, and 28 DEGs and corresponding downregulation of 187, 12, 20, and 25 DEGs, respectively (Table S1). Compared to BP1, GO enrichment analysis (Tables S4) revealed that DEGs in BP2 enriched in transmembrane transport (GO:0055085), with 34 DEGs including sugar transporters (*HXT13* and *HXT17*),^29^ amino acid transporters (*AGP1* and *AGP2*),^30^^,^^31^ and ion transporters (*PHO84*, *ENA1*, *ENA2*, and *ENA5*).^32^^,^^33^ rRNA processing (GO:0006364) and ribosome biogenesis (GO:0042254) were upregulated, indicating active protein synthesis. For CCs, plasma membrane (GO:0005886) contained 65 DEGs related to transporters, corroborated by strong enrichment in cell periphery (GO:0071944). Compared to BP3, BP4 maintained enrichment in transmembrane transport (GO:0055085) and transmembrane transporter activity (GO:0022857) (Figures 3C and 3D; Table S4). Upon glucose depletion, minimal transcriptional variations occurred under high ethanol concentrations. Throughout fermentation, DEGs were predominantly concentrated in molecular function (Figure 3E). KEGG enrichment analysis confirmed the transcriptional stability of BPCs compared to FPCs (Figures 3F and 3G; Table S5). BP2 contained 431 DEGs relative to BP1 (Table S1). The transcriptome results indicated that carbon metabolism DEGs were downregulated while amino acid metabolism DEGs were upregulated, promoting biosynthesis of histidine (*HIS4*), cysteine (*MET17*), methionine (*MET6*), valine (*ILV2*), phenylalanine (*ARO1* and *ARO4*), lysine (*LYS20* and *LYS2*), and arginine (*ARG56*, *ARG8*, *ARG1*, and *ARG4*), redirecting metabolism toward amino acid biosynthesis (Figure 3K). BP3 and BP4 showed minimal variations compared to BP2. BP5, collected 1 h post-fermentation, displayed upregulated DEGs concentrated in glyoxylate cycle pathways (Figure 3K). Throughout the fermentation process, glycolytic intensity remained transcriptionally stable.

Following the demonstration of transcriptomic stability in BIF, BPCs and L1 cells were analyzed to provide complementary insights. Three samples of L1 cells (B2I1, B4I1, and B5I1) yielded only 105 DEGs (Figure 3H), with 24 and 41 upregulated DEGs and 25 and 24 downregulated DEGs during the BIF process, respectively (Table S1). Comparative analysis revealed that B4I1 DEGs were strongly enriched in CC (GO:0005575) and fungal-type cell wall (GO:0009277) relative to B2I1 (Table S6), indicating cell-wall remodeling in response to ethanol stress, alongside extracellular region modifications (Figure 3I). Upon ethanol exposure post-fermentation, B5I1 DEGs were enriched in Ty elements GO terms (Table S6), including viral translational frameshifting (GO:0075523), retrotransposition (GO:0032197), and retrotransposon nucleocapsid (GO:0000943) (Figure 3J). KEGG pathway analysis identified none clustered pathway across the temporal samples (Figure 3K). Notably, *FLO11* exhibited significant upregulation in B5I1 compared to B4I1 (Table S7).

### Biofilm cells underwent a process of separation and re-adhesion during the fermentation process

Given the transcriptomic stability observed in both planktonic cells and L1 cells during BIF, we investigated the relationships between these populations and their temporal variations. Three comparative groups were analyzed: SVG1 (B2I1 vs. BP2), SVG2 (B4I1 vs. BP4), and SVG3 (B5I1 vs. BP5), yielding 1,290 total DEGs with 425, 418, and 654 upregulated DEGs and 188, 177, and 353 downregulated DEGs in B2I1, B4I1, and B5I1, respectively (Table S1).

**Figure 4:**
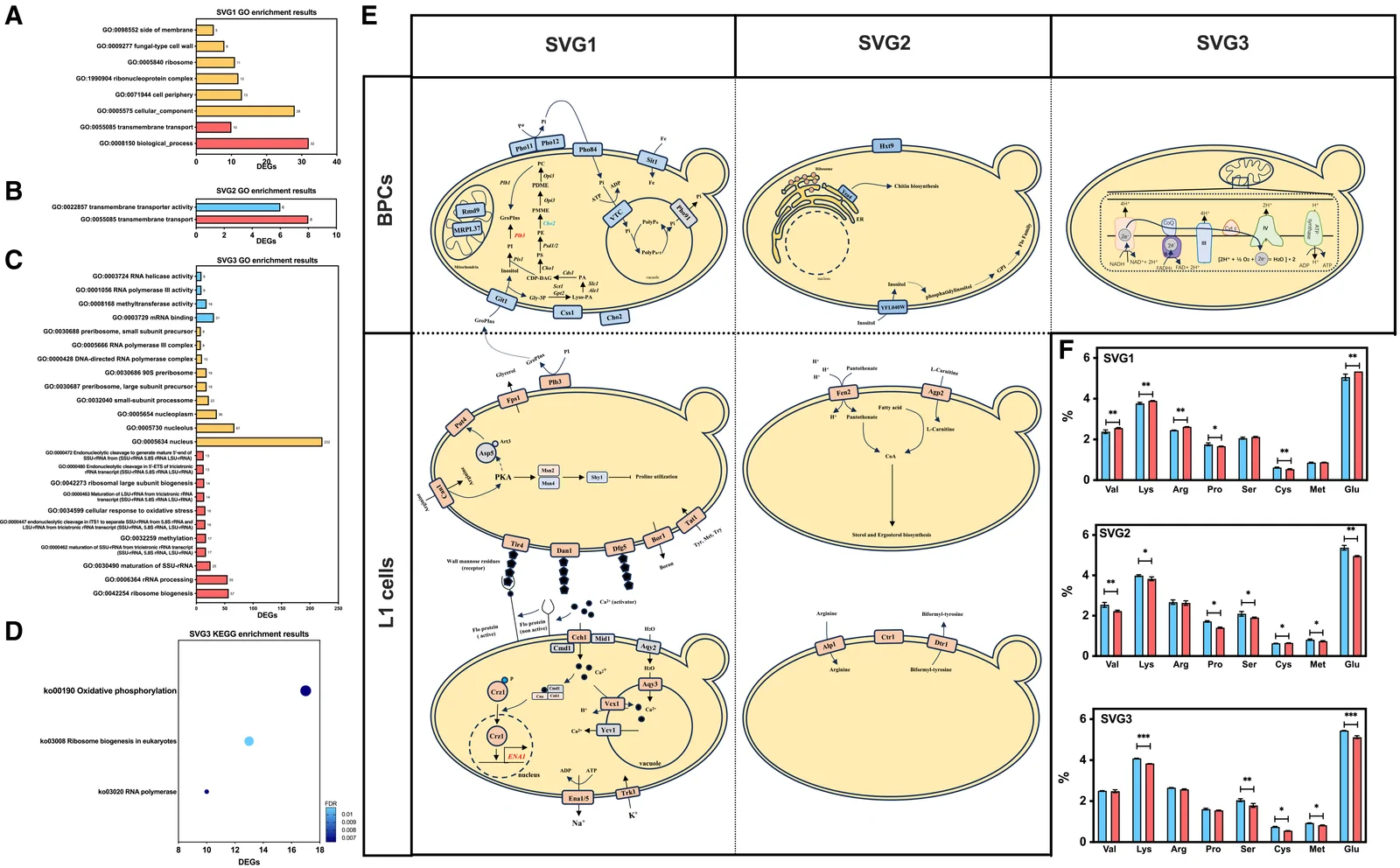
Transcriptome analysis for cell communications between BPCs and L1 cells (A–C) GO (the blue bar: biological progress; the yellow bar: cellular component; the red bar: molecular function) and (D) KEGG enrichment analysis in significant varied genes between planktonic cells and L1 cells at 2 h (A-SVG1), 4 h (B-SVG2), and 5 h (C-SVG3); (E) DEGs distributed in cell communications between BPCs and L1 cells (blue background indicates upregulation in planktonic cells, red background indicates upregulation in L1 cells, and gray background indicates no significant difference between the BPCs and L1 cells). (F) The levels of amino acids in BPCs and L1 cells in SVG1, SVG2, and SVG3 (blue bar represented amino acids levels in BPCs and red bar represented amino acids levels in L1). Data are represented as mean ± SEM; ∗*p* < 0.05, ∗∗*p* < 0.01, ∗∗∗*p* < 0.001, ∗∗∗∗*p* < 0.0001; one-way ANOVA (*n* = 3 biological replicates per group). SVG1, SVG2, and SVG3, significant varied genes between planktonic cells and L1 cells at 2, 4, and 5 h, respectively. BPCs, BIF’s planktonic cells including BP1, BP2, BP3, BP4, and BP5; L1, the first layer of the biofilm cells (the biofilm cells obtained after the first vortex oscillation stage (60 W, 15 s) of the carrier at batch 8 including B2I1, B4I1, and B5I1); GroPIns, glycerophosphoinositol; Lyso-PA, lysophosphatidic acid; PA, phosphatidic; CDP, cytidine diphosphate; DAG, diacylglycerol; PS, phosphatidylserine; Gly-3P, Glycerol-3-phosphate; PI, phosphatidylinositol; PC, phosphatidylcholine; PMDE, phosphatidyldimethylethanolamine; PE, phosphatidylethanolamine; PMME, phosphatidylmonomethylethanolamine; Val, valine; Lys, lysine; Arg, arginine; Pro, proline; Ser, serine; Cys, cysteine; Met, methionine; Glu, glutamate.

SVG1 exhibited significant enrichment in ribosome biogenesis (GO:0005840), transmembrane transport (GO:0055085), and cell periphery (GO:0071944) (Figure 4A; Tables S8 and S9). B2I1 cells showed upregulation of transporters including boron transporter (*BOR1*),^34^ amino acid transporters (*TAT1* and *CAN1*),^35^^,^^36^ and metal ion transporters (*CCH1*, *TRK1*, and *ENA5*).^37^^,^^38^

As glucose consumption (SVG2, Figure 4B; Tables S8 and S9), B4I1 cells prioritized transmembrane transporter activity (GO:0022857) with upregulated DEGs including *ALP1*, *FEN2*, *HNM1*, and *DTR1*, facilitating nutrient uptake under physical constraints.

In SVG3 (Figure 4C; Tables S8 and S9), B5I1 cells exhibited enrichment in rRNA processing (GO:0006364) and RNA polymerase activity (ko03020). BP5 showed upregulated DEGs related to ATP synthesis (Figure S3), while B5I1 cells preferentially expressed DEGs associated with ATP utilization (Figure S4).

### The outer shield: L1 cells sacrifice mitochondrial function for stress tolerance

**Figure 5:**
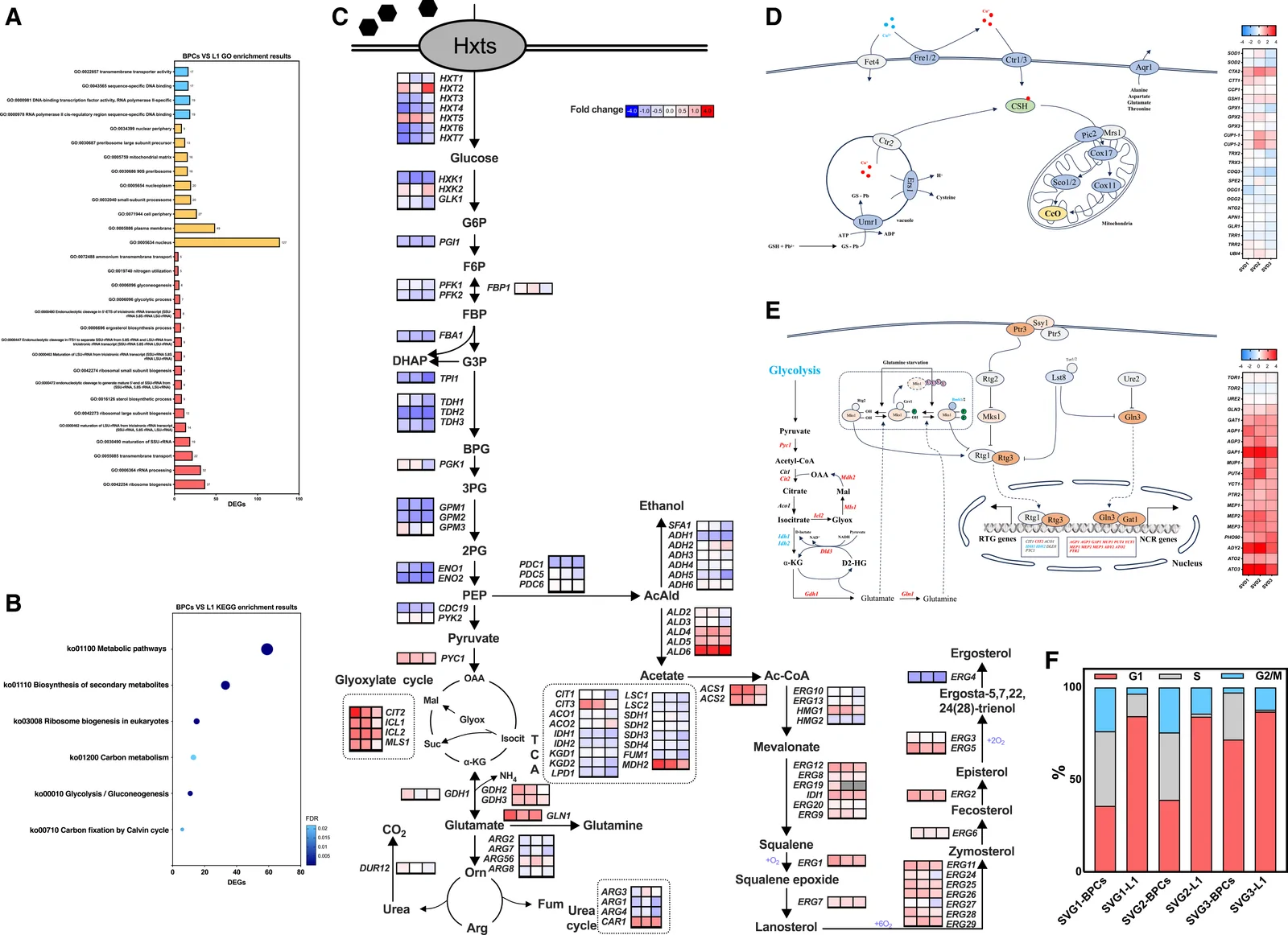
Transcriptome analysis of BPCs and L1 in spatial dimensions GO (the blue bar: biological progress; the yellow bar: cellular component; the red bar: molecular function) (A) and KEGG (B) enrichment analysis between BPCs and L1 cells; (C) difference in gene expressions related to carbon metabolism (glycolysis, pyruvate metabolism, TCA, glyoxylate cycle) and nitrogen metabolism (glutamate biosynthesis, arginine biosynthesis, and ergosterol biosynthesis). The sample names from left to right are SVG1, SVG2, and SVG3. (D) BPCs preferred copper uptake to maintain ETC activity (blue: upregulated in BPCs, red: upregulated in L1, and gray: non-significant change between the BPCs and L1 cells); (E) L1 integrated retrograde (RTG) signaling pathway and nitrogen catabolite repression (NCR) genes to cope with nitrogen deficient (blue: upregulated in BPCs, red: upregulated in L1, and gray: non-significant change between the BPCs and L1 cells); (F) Cell cycle distributions of BPCs and L1 cells in SVG1, SVG2, and SVG3. Data are represented as mean ± SEM (*n* = 3 biological replicates per group). BPCs, BIF’s planktonic cells including BP1, BP2, BP3, BP4, and BP5; L1, the first layer of the biofilm cells (the biofilm cells obtained after the first vortex oscillation stage (60 W, 15 s) of the carrier at batch 8 including B2I1, B4I1, and B5I1); HXTs, hexose transporters; G6P, glucose-6-phosphate; F6P, fructose-6-phosphate; FBP, fructose-1,6-bisphosphate; G3P, glyceraldehyde 3-phosphate; BPG, 1,3-bisphosphoglycerate; 3 PG, 3-phosphoglycerate; 2 PG, 2-phosphoglycerate; PEP, phosphoenolpyruvate; DHAP, dihydroxyacetone phosphate; AcAld, acetaldehyde; Ac-CoA, Acetyl-CoA; OAA, oxaloacetate; Iso, isocitrate; α-KG, α-ketoglutarate; Suc, succinate; Mal, malate; Orn, ornithine; Arg, arginine; Fum, fumarate.

During BIF process, 292 DEGs were also shared between BPCs and L1 cells in temporal dimensions. In Figure 5A, L1 cells showed strong enrichment in ribosome (GO:0042254) and rRNA processing (GO:0006364) (Table S10). These processes are critical for maintaining ribosomal assembly under adhesion-induced stress. Also, DEGs of L1 upregulated in nucleolus (GO:0005634) and small-subunit processome (GO:0032040), reflecting active rRNA maturation. Additionally, transmembrane transport (GO:0055085) was upregulated suggesting enhanced nutrient uptake in L1, excluding *ERS1*, *AQR1*, and *VMR1*.^39^^,^^40^^,^^41^ DEGs in sterol biosynthesis process (GO:0016126) were also upregulated. For KEGG enrichment analysis (Figure 5B; Table S11), L1 cells significantly preferred to protein biosynthesis (ko03008 Ribosome biogenesis in eukaryotes) but downregulated DEGs in glycolysis/gluconeogenesis (ko00010) (Figure 3C).

## Spatial division of labor: A gradient from metabolic defense to active proliferation

**Figure 6:**
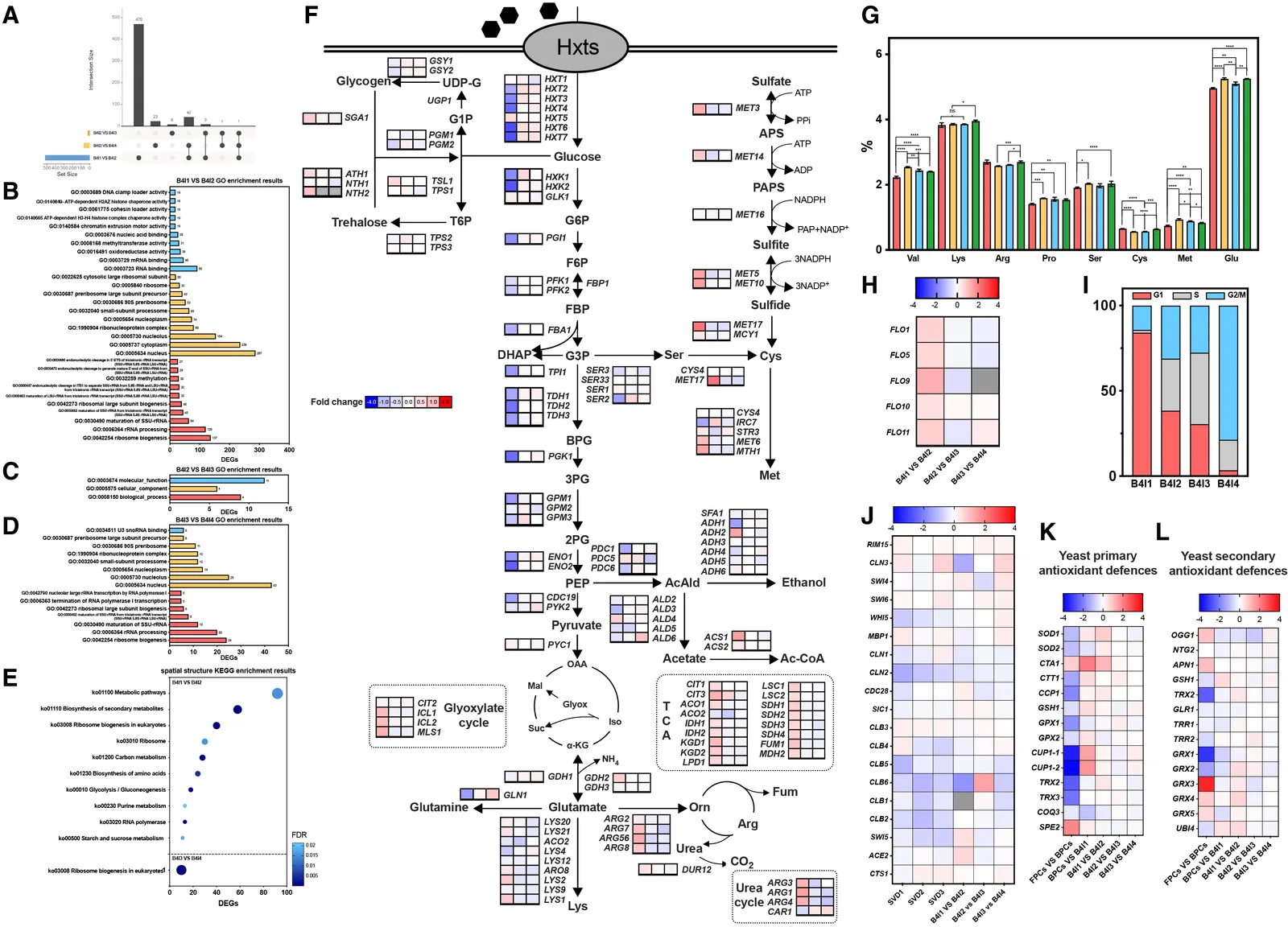
Transcriptome analysis of biofilm in spatial dimensions (A) Overview of DEGs in three groups (B4I2 vs. B4I1, B4I3 vs. B4I2, and B4I4 vs. B4I3); GO (the blue bar: biological progress; the yellow bar: cellular component; the red bar: molecular function) and KEGG (E) enrichment analysis in B4I2 vs. B4I1 (B), B4I3 vs. B4I2 (C), and B4I4 vs. B4I3 (D); (F) difference in gene expressions related to carbon metabolism (glycolysis, glycogen biosynthesis, trehalose biosynthesis, pyruvate metabolism, TCA, glyoxylate cycle) and nitrogen metabolism (cysteine and methionine biosynthesis, glutamate biosynthesis, lysine biosynthesis, and arginine biosynthesis). The sample names from left to right are B4I2 vs. B4I1, B4I3 vs. B4I2, and B4I4 vs. B4I3. (G) The levels of amino acids in biofilms (red bar represented amino acid levels in B4I1, yellow bar represented amino acid levels in B4I2, blue bar represented amino acid levels in B4I3, and green bar represented amino acids levels in B4I4). Data are represented as mean ± SEM; ns, not significant; *p* > 0.05, ∗*p* < 0.05, ∗∗*p* < 0.01, ∗∗∗*p* < 0.001, ∗∗∗∗*p* < 0.0001; one-way ANOVA (*n* = 3 biological replicates per group); (H) Difference in gene expressions related *FLO Family* in biofilms; (I) Data are represented as mean ± standard deviation. Cell cycle distributions in B4I1, B4I2, B4I3, and B4I4; (J) difference in gene expressions related cell cycle progression in B4I1, B4I2, B4I3, and B4I4; difference in the expressions of genes related to the primary (K) and secondary (L) antioxidant defences. B4I1, B4I2, B4I3, and B4I4, the biofilm cells obtained after the first, second, third, and fourth vortex oscillation stage (60 W, 15 s) of the carrier, respectively, at 4 h at batch 8; HXTs, hexose transporters; G6P, glucose-6-phosphate; F6P, fructose-6-phosphate; FBP, fructose-1,6-bisphosphate; G3P, glyceraldehyde 3-phosphate; BPG, 1,3-bisphosphoglycerate; 3 PG, 3-phosphoglycerate; 2 PG, 2-phosphoglycerate; PEP, phosphoenolpyruvate; G1P, glucose-1-phosphate; UDP-G, UDP glucose; T6P, trehalose-6-phosphate; DHAP, dihydroxyacetone phosphate; Ser, serine; Cys, cysteine; Met, methionine; APS, adenylyl sulfate; PAPS, 3-phosphoadenylyl sulfate; AcAld, acetaldehyde; Ac-CoA, acetyl-CoA; OAA, oxaloacetate; Iso, isocitrate; α-KG, α-ketoglutarate; Suc, succinate; Mal, malate; Lys, lysine; Orn, ornithine; Arg, arginine; Fum, fumarate.

To elucidate the spatial functional organization, we analyzed gene expression profiles across the biofilm depth, from the outer surface (L1: B4I1) to the inner core (L3: B4I3 and B4I4). A clear functional gradient was observed, indicating a strategic division of labor. Overall, a total of 554 DEGs were identified among the four samples (Figure 6A), with 104, 14, and 55 upregulated genes and 418, 6, and 12 downregulated genes across spatial comparisons (B4I2 vs. B4I1, B4I3 vs. B4I2, and B4I4 vs. B4I3), respectively (Table S1).

At the outer surface (L1:B4I1), comparative analysis with L2 (B4I2), GO enrichment analysis (Table S12) revealed that DEGs involved in ribosome biogenesis (GO:0042254), Small Subunit Ribosomal RNA (SSU-rRNA) processing (GO:0030490), nucleolus (GO:0005730), and 90S preribosome (GO:0030686) were upregulated in L1 (B4I1), indicating preparation for protein biosynthesis and metabolic activation^41^ (Figure 6B). Also, L1 showed that 30 DEGs were identified in methylation processes (GO:0032259) and methyltransferase activity (GO:0008168), with 26 DEGs significantly upregulated in L1 and 5 genes (*MET6*, *TMT1*, *CHO2*, *MHT1*, and *YNL092W*) downregulated. Transcriptomic analysis revealed distinct metabolic stratification across the biofilm layers. The outermost layer (L1) exhibited a broad activation of methyltransferases, including histone methyltransferases (*HMT1*, *BMT2*, *BMT5*, *BMT6*, and *HPM1*), RNA methyltransferases (*NOP1*, *NOP2*, *RRP8*, *TRM1*, *TRM2*, *TRM8*, *TRM10*, *TRM11*, and *TRM44*), and others (*EFM2*, *SPB1*, *RRP8*, *RMT2*, *UPA1*, *UPA2*, *AIR1*, *GCD14*, *EMG1*, *SHM2*, and *ERG11*), suggesting a high level of epigenetic regulation and protein synthesis priming. KEGG enrichment analysis demonstrated a marked upregulation of carbon metabolism (ko00010 glycolysis/gluconeogenesis, ko01200 carbon metabolism, and ko00230 purine metabolism, and ko00500 starch and sucrose metabolism) and amino acid metabolism (ko01230 biosynthesis of amino acids) (Figure 6E) (Tables S12 and S13).

In the middle cells (L2), cell cycle analysis (Figure 6I) showed that G2/M phase gradually becomes increasingly enriched as the depth of the biofilm increase. This observation was supported by transcriptional data, specifically the upregulation of *ACE2* and downregulation of *CLB6* and *CLN3* (Figure 6J), indicating G2 phase enrichment in L2 compared to L1. All *FLO* genes were upregulated in L2 but downregulated in L3 (B4I3 and B4I4) (Figure 6H).

In the inner cells (L3: B4I3 and B4I4), the B4I3 vs. B4I2 comparison yielded only 20 DEGs, including *CLB6*, resulting in increased S-phase cell populations (Figures 6I and 6J). In the B4I4 vs. B4I3 comparison, DEGs were enriched in ribosome biogenesis (GO:0042254), rRNA processing (GO:0006364), SSU-rRNA maturation (GO:0030490), SSU-rRNA maturation from tricistronic transcript (GO:0000462), ribosomal large subunit biogenesis (GO:0042273), RNA polymerase I transcription termination (GO:0006363), and nucleolar large rRNA transcription (GO:0042790), indicating active cell cycle progression (Figures 6C and 6D) (Table S12). KEGG analysis showed upregulation of ribosome biogenesis pathways (ko03008), promoting cell cycle progression and G2/M phase enrichment (Figure 6I)^42^ (Table S13).

Regarding amino acid profiles, the spatial distribution of eight representative amino acids was analyzed to evaluate the metabolic status of different biofilm layers (Figure 6G). These amino acids were categorized into three functional groups: (1) glycolytic diversion indicators (Ser, Cys, and Met); (2) the pyruvate metabolism indicator (Val); and (3) TCA cycle (Tricarboxylic acid cycle)-downstream metabolites (Glu, Lys, Arg, and Pro). Figure 6G revealed that the lowest levels of almost all measured amino acids were recorded in the outermost layer (L1, B4I1). Accumulation levels then surged to a peak in the middle layer (L2, B4I2). As the depth increased into the innermost layers (L3, comprising B4I3 and B4I4), the concentrations of these amino acids exhibited a gradual decline compared to the L2 peak, although they generally remained higher than those in L1. Notably, Glu and Pro followed this “increase-then-decrease” trajectory most prominently, suggesting that the metabolic diversion from central carbon metabolism is most intense in the intermediate region of the biofilm.

## Discussion

### Enhanced fermentation performance and physiological robustness in BIF

Bioethanol production has traditionally relied on FCF; however, BIF has recently demonstrated superior fermentation performance.^42^^,^^43^^,^^44^^,^^45^^,^^46^^,^^47^ Consistent with previous findings, our study confirmed that BIF significantly outperformed FCF, especially in terms of productivity (Figure 1C). The rapid fermentation rate in BIF generates substantial heat over a short period, exposing cells to greater thermal stress than in FCF. Both thermal and ethanol stress are known to induce trehalose synthesis as a protective mechanism,^43^^,^^44^ which explains the elevated trehalose levels observed in BIF (Figure 1G). Furthermore, glycogen, a critical reserve carbohydrate for fermentation efficacy^45^ was significantly accumulated in BPCs and outer biofilm cells (BCs, L1) (Figure 1H) conferring yeast cells with enhanced survival and reproductive advantages.^46^ Unlike in some embedded immobilization systems where glycogen accumulation is linked to cell-cycle arrest^42^^,^^47^ in the G0 phase, our data suggest that in BIF, these reserves support active growth, metabolism, and reproduction within the biofilm architecture. Physiological analysis further revealed that BPCs and L1 cells exhibited lower apoptosis rates and reduced mortality compared to FPCs (Figures 1D and 1F), indicating that the biofilm lifestyle confers enhanced viability and robustness essential for continuous batch fermentation.

### Distinct temporal transcriptional landscapes: Survival-driven FCF vs. stable BIF

In FCF, gene expression patterns are predominantly driven by the struggle for survival against deteriorating environmental conditions. At FP2 stage (glucose: 26.36 g/L; ethanol: 7.20 g/L), cells benefited from alleviated substrate inhibition. Transcriptionally, the rate-limiting glycolytic enzymes hexokinase (*HXK1*, *HXK2*, *GLK1*), phosphofructokinase (*PFK1*, *PFK2*), and pyruvate kinase (*CDC19*, *PYK2*) showed stable expression, while DEGs promoting glycogen (*PGM2*, *GSY1*, *GSY2*), β-1,3-glucan (*GSC2*), and trehalose (*TPS1*, *TSL1*, *TPS2*) accumulation was upregulated. To balance the glycolytic flux inhibited by ATP and NADH (Nicotinamide adenine dinucleotide) accumulation,^48^ cells upregulated sulfur assimilation pathways. This process consumes ATP and NADH,^49^ facilitating the synthesis of sulfur-containing amino acids (cysteine and methionine) essential for protein folding, assembly, and stability.^50^ As glucose levels declined less 5 g/L, ethanol production increased while cell growth decelerated (Figure 1A). FPCs shifted carbon flux from glycolysis toward the biosynthesis of L-valine, arginine, and L-lysine to sustain growth under high ethanol stress.^51^^,^^52^^,^^53^ Ultimately, upon glucose depletion, cells activated the glyoxylate cycle and gluconeogenesis (*FBP1*) to maintain glucose homeostasis via the catabolism of storage compounds (glycogen and trehalose). Overall, in FCF, FPCs under nutrient-rich conditions, cells actively accumulate resources for growth and proliferation through sulfur assimilation and sulfur-containing amino acid biosynthesis for protein synthesis, accompanied by energy storage compound accumulation (glycogen and trehalose). Glycolysis-derived ATP and NADH accumulation inhibit glycolytic flux and glucose uptake; therefore, maintaining ATP and NAD^+^ (Nicotinamide adenine dinucleotide)/NADH balance through ethanol production is crucial for maximizing glycolytic energy yield. Under harsh conditions (high cell density, elevated ethanol concentrations, and carbon limitation), cells initiate protective mechanisms against ethanol toxicity. Upon further carbon source depletion, cells utilize storage compounds and activate gluconeogenesis to maintain glucose homeostasis.

In sharp contrast, BIF exhibited remarkable transcriptional stability over time. Only 517 DEGs were identified in BPCs throughout the process, with the majority occurring during the initial transition (430 DEGs from BP1 to BP2). While glycolytic gene expression remained constant, carbon flux was diverted toward the biosynthesis of L-valine (*ILV2*), chorismate (*ARO1* and *ARO4*), and L-histidine (*HIS4*).^54^^,^^55^^,^^56^ Unlike FCF, FPCs accumulated L-arginine while downregulating glutamine synthetase (*GLN1*) (Figure S5). As fermentation progressed, BPCs consistently enriched TCA or glyoxylate cycle pathway. Notably, BPCs showed no preference for trehalose biosynthesis as a protective mechanism; instead, arginine accumulation served as the dominant factor for environmental resistance. L1 cells (B2I1, B4I1, and B5I1) on the biofilm exterior were directly exposed to ethanol stress yet exhibited only 105 DEGs temporally, which showed no significant preference for carbon or amino acid metabolism, indicating greater transcriptional stability and reduced environmental sensitivity in outer BCs (L1 cells).

### Metabolic adaptation in outer biofilm layers: Mitochondrial dysfunction and retrograde signaling

Spatially, the outer layer cells (L1 cells) interact directly with the harsh fermentation environment, yet they exhibited fewer temporal transcriptional changes than BPCs, indicating a buffered sensitivity. However, significant metabolic remodeling was observed when comparing L1 to BPCs (SVG1, SVG2, and SVG3). DEGs in SVG1 and SVG2 were predominantly concentrated in membrane-related processes including transmembrane transport (GO:0055085), sporulation (GO:0030435), cell periphery (GO:0071944), fungal-type cell wall (GO:0009277), and membrane-associated functions (GO:0098552) (Figure 4A). In SVG1 (Figure 4E), BPCs activated phosphate and glycerophosphoinositol (GroPIns) transport. L1 cells activated Ca^2+^ uptake to balance equilibrium and inactive Flo protein, preparing for cell release. L1 cells also exhibited specific amino acid transport preferences for slow growth and inhibition biofilm formation. Specifically, BPCs upregulated *PHO11*, *PHO12*, and *PHO84* for phosphate transport and vacuolar storage via vacuolar transporter chaperone complex,^57^^,^^58^ and *GIT1* for glycerophosphoinositol uptake, preparing for cell release. *CCH1* transported Ca^2+^ into L1 cells for vacuolar storage via *VCX1*,^59^^,^^60^ while *AQY3* facilitated H_2_O uptake to balance Ca^2+^ concentrations.^61^ Intracellular Ca^2+^ dephosphorylated Crz1p, enabling nuclear translocation and *ENA1* transcription,^62^^,^^63^ which exports sodium ions for cellular detoxification.^64^^,^^65^ *TRK1* maintained potassium homeostasis.^66^ On the other hand, *TIR4*, *DAN1*, and *DFG5* encoded cell wall mannoglycoproteins targeted by Flo proteins for cell-cell adhesion.^67^^,^^68^ In addition, cells exhibited specific amino acid transport preferences. *CAN1* overexpression downregulated biofilm formation while facilitating arginine transport and inhibiting proline assimilation through *PUT4* endocytosis.^69^^,^^70^ *TAT1* transported tryptophan, tyrosine, and methionine,^35^^,^^71^ with decreased intracellular tryptophan resulting in growth retardation.^72^ *BOR1* overexpression exported boron, causing deficiency and growth deceleration.^73^ Boron and tryptophan cooperatively regulated immobilized cell growth. Additionally, *SEG1* overexpression improved stress tolerance,^74^^,^^75^ while enhanced ascospore synthesis (*SPO24*, *OSW2*) and assembly (*SPS2*, *SPS100*) were observed.^66^^,^^76^^,^^77^ In SVG2 (Figure 4E), BPCs prepared re-adhesion to L1 cells, and L1 cells increase biofilm formation abilities. BPCs overexpressed *YEA4*, required for chitin biosynthesis and biofilm characteristics.^78^ *YFL040W* upregulation facilitated inositol transport,^79^ a phosphatidylinositol precursor essential for Flo family GPI (Glycosylphosphatidylinositol)-anchor assembly.^80^ L1 cells overexpressed *FEN2*, *AGP2*, *ALP1*, *DTR1*, and *CTR1*. *FEN2* and *AGP2* transported pantothenate and L-carnitine for CoA accumulation supporting sterol and ergosterol biosynthesis,^31^^,^^81^^,^^82^ with most related biosynthetic genes upregulated (Figure 5C). To circumvent *CAN1*-mediated biofilm inhibition,^69^ L1 cells utilized ALP1p for arginine assimilation,^30^ maintaining biofilm stability. *CTR1* activation by MAC1 also promoted biofilm formation.^83^ In SVG3 (Figure 4E), DEGs were enriched in oxidative stress response (GO:0034599) beyond transcription and protein synthesis. L1 cells utilized *MTL1* and *YBL055C* for oxidative stress response,^79^^,^^84^ indicating biofilm-mediated protection through enhanced physical barriers. BPCs integrated diverse functions including oxidative stress response (*AHP1*, *TSA1*, *GRX1*, *GRX2*, *HBN1*, *YHB1*), energy metabolism (*ATP1*, *ATP2*, *ATP5*, *ATP7*, *ATP16*, *ATP18*, *ATP20*, *COX12*), ion homeostasis and detoxification (*ATX1*, *SCO1*, *VMA15*, *VMA16*, *PMA1*, *SMF1*), DNA damage repair (*DUN1*, *HSP31*), cellular homeostasis (*GAD1*), and osmoregulation (*GCY1*, *LOT6*). Temporally, during BIF, BPCs underwent dispersion followed by re-adhesion processes. SVG1 demonstrated L1 cells transporting Ca^2+^ and inactivating Flo proteins, resulting in cell dispersion, while planktonic cells accumulated phosphate and transported glycerophosphoinositol for release preparation. SVG2 showed BPCs preparing for biofilm re-adhesion. Upon glucose depletion and ethanol exposure (SVG3), both BPCs and L1 cells integrated limited resources to respond to environmental stress through distinct metabolic pathways.

Following analysis of DEGs between BPCs and L1 cells in temporal dimensions, we examined spatial dimension DEGs by screening 292 DEGs showing temporal stability. BPCs preferentially maintained glycolytic activity (Figure 5C) and ensured electron transport chain (ETC) function through cuprous ion transport (Figure 5D). Although the gene expression of L1 in the glycolysis and ethanol fermentation process is lower than that of BPCs, it is superior to that of FCF (Figure S9). L1 cells upregulated sterol and ergosterol biosynthesis genes (Figure 5C) to adjust membrane fluidity and maintain cellular integrity against ethanol stress.^85^ In biofilms, cell stacking further limited oxygen and nutrient diffusion, leading to mitochondrial dysfunction in L1 cells. This constraint imposes a metabolic bottleneck of the TCA cycle, evidenced by the downregulation of *IDH1/2*, which decreases α-ketoglutarate (α-KG) levels and subsequently reduces glutamate and ATP production while accumulating reactive oxygen species (ROS) (Figures S6–S8).^86^ To compensate for this “glutamate crisis,” L1 cells activated the mitochondrial retrograde (RTG) signaling pathway.^86^ The glutamate deficiency and downregulated *BMH1* likely relieved the inhibition of the Bmh1p-Mks1p complex, allowing Mks1p to interact with Rtg2p.^87^ This interaction facilitates the nuclear translocation of the Rtg1p-Rtg3p complex, activating target genes such as *CIT2* (peroxisomal citrate synthase, hallmark of robust retrograde response), *DLD3* (2-hydroxyglutarate transhydrogenase) and *PYC1* (pyruvate carboxylase), expression, further activating the glyoxylate cycle.^88^^,^^89^^,^^90^^,^^91^^,^^92^ This anaplerotic mechanism replenishes α-KG to rescue glutamate biosynthesis.^93^ Simultaneously, the downregulation of *LST8* relieved the repression of *GLN3*, leading to the upregulation of Nitrogen Catabolite Repression (NCR) genes to enhance extracellular nitrogen uptake.^94^ Thus, L1 cells survive by integrating glycolysis for ATP, the RTG pathway for anabolic precursors,^86^ and antioxidant defense (*CTA1*, *GSH1*) to mitigate ROS damage.^95^

### Spatial division of labor: Stratification of structure and proliferation

The spatial architecture of *S. cerevisiae* biofilms in this study was discretized into three representative layers (L1-L3) using a sequential detachment approach. This sampling strategy is predicated on the physical principle that peripheral cells, being less constrained by the extracellular polymeric substance matrix, are released more readily than those anchored in the basal regions near the carrier. While these “layers” represent a simplified model of a continuous biological gradient, the distinct transcriptomic profiles—ranging from high metabolic activity in L1 to resource-conservation strategies in L3—confirm that this methodology effectively captures the intrinsic spatial heterogeneity of the biofilm.

Based on this stratified framework, a clear functional division of labor was observed as depth increased. In biofilms, L1 cells suffered ETC dysfunction, decreasing glutamate and ATP while increasing ROS accumulation and activating the RTG pathway. Although peripheral cells (L1) downregulated glycolytic intensity transcriptionally, activity remained significantly enhanced compared to FCF (Figure S9). Glutamate deficiency dephosphorylated Mks1p, activating Rtg1p-Rtg3p nuclear translocation and RTG gene activation. Cells activated the RTG pathway to maintain α-ketoglutarate levels and rescue glutamate production while upregulating primary ROS defense mechanisms. In the middle layer (L2), glycolytic activity was further attenuated, and metabolism shifted toward the glyoxylate cycle, supported by *ADH1* downregulation and *ADH2* upregulation for acetyl-CoA production^96^ (Figure 6F). Uniquely, L2 cells upregulated all *FLO* genes showed upregulation (Figure 6H). For amino acid metabolism, L2 decreased *GLN1* expression as glutamate restriction was alleviated (Figures 6F and 6G). DEGs enriched in arginine and cysteine biosynthesis were upregulated (Figure 6F). Zara et al. reported that 10 mM L-arginine and L-cysteine inhibited biofilm formation in wild-type yeast by reducing *FLO11* expression.^97^ Moreover, L2 decreased arginine and cysteine content while upregulating sulfate metabolism, potentially contributing to cysteine or Methionine accumulation. However, downregulated ribosome biogenesis, rRNA processing and maturation DEGs, combined with upregulated methylation DEGs, indicated cysteine was utilized for methionine biosynthesis rather than protein synthesis, supported by upregulated *MET6* and *MHT1* (Figure 6G). Similarly, methionine biosynthesis genes were upregulated in *Candida albicans* biofilms,^98^ and methionine limitation impaired biofilm formation in *E. coli*.^99^ Overall, L2 cells upregulated all *FLO* genes and specifically modulated sulfur-amino acid partitioning-favoring methionine accumulation over cysteine as confirmed by the spatial metabolite profiles. This metabolic redirection provides robust evidence that the L2 layer serves as the functional structural scaffold of the biofilm, prioritizing matrix stability. In the innermost layer (L3), the gene expression profile was dominated by ribosome biogenesis and cell cycle progression. B4I3 yielded only 20 DEGs, with B-type cyclin CLB6p upregulation supporting START processing and active cell cycle progression within biofilms. Deeper cells (B4I4) were enriched for upregulated DEGs in ribosome biogenesis and RNA processing and maturation, indicating that the core of the biofilm acts as a protected niche for proliferation. For cell cycle change, compared to BPCs, L1 downregulated cell cycle genes (*WHI5*, *CLN1*, *CLN2*, *CLB5*, *CLB6*, *CLB1*, *CLB2*, *SWI5*), indicating BPCs showed minimal cell cycle variations. BCs from internal to outer regions displayed complete cell cycle progression (Figures 6I and 6J). It is hypothesized that active internal cells may migrate outward similar to *Vibrio cholerae* biofilms.^100^

This spatial organization resembles the differentiation seen in colony biofilms. In colony biofilms, colonies diverged into distinct subpopulations: U cells (upper regions) and L cells (lower regions). U cells are long-lived with unique properties regulated by RTG pathways, activating amino acid biosynthesis and reducing mitochondrial translation.^23^ Similar to U cells, outer cells (L1) upregulated RTG pathways and amino acid biosynthesis. L cells upregulated oxidative phosphorylation and gluconeogenesis genes,^22^ while biofilm internal cells preferred protein biosynthesis and active cell cycle progression. Previous studies have shown that the cells within the biofilm undergo an active cell cycle progress.^42^^,^^101^ The cell cycle progression and ribosome synthesis for B4I3 and B4I4 are both in an active state. Here, we classify them as innermost cells (L3), which were spatially arranged with upregulating *CLN3* (G1/S phase preference), and *CLB6* (S phase entry), and L2 upregulating *SWI5* and *ACE2* (G2/M phase). Collectively, BIF achieves a “reproduction-metabolism decoupling”: outer cells maximize glycolysis and endure stress; middle cells maintain structure; and inner cells drive population expansion.

Despite the demonstrated advantages of BIF, the genetic basis of its carbon flux rearrangement has remained elusive. Most research has focused on strain engineering rather than the intrinsic heterogeneity of the biofilm. Ideally, investigating this spatiotemporal heterogeneity requires precise cell sampling from specific depths. However, this is practically challenging due to the intricate, rough surface of the cotton fiber carriers and the hydrodynamic shear of fermentation. Therefore, it should be acknowledged that the “spatial layers” defined in this study are inferred from differential cell adhesion forces (sequential detachment) rather than *in situ* imaging. Given the irregular topography of fibrous carriers, a standardized physical definition of biofilm layers is yet to be established. Nevertheless, our approach provides a pioneering conceptual framework for understanding metabolic stratification in industrial biofilms. While this model simplifies the complex architecture, it successfully captures the critical phenomenon of reproduction-metabolism decoupling. We anticipate that future advancements, particularly in single-cell spatial transcriptomics, will allow for a high-resolution refinement of this model to further optimize biofilm-immobilized fermentation technologies. The functional division of labor in *S. cerevisiae* biofilms is characterized by a strategic trade-off between structural maintenance and reproductive potential. Our findings suggest that the metabolic specialization of the middle layer (L2) in structural scaffolding and the innermost layer (L3) in proliferation is not a series of independent responses, but a cooperative population-level strategy. By compartmentalizing stress response and reproduction, the biofilm minimizes the global metabolic burden and ensures the preservation of a viable cell reservoir even under harsh industrial conditions, thereby achieving long-term evolutionary and functional stability.

Moreover, the spatial heterogeneity of amino acid accumulation provides a metabolic roadmap for the “division of labor” with the *S. cerevisiae* biofilm. In the outermost layer (L1), the minimal accumulation of glycolytic-diversion amino acid (Ser, Cys, and Met) and TCA-downstream metabolites (Glu and Pro) suggests that carbon flux is strictly prioritized for the glycolytic pathway. This “lean” metabolic profile indicates that L1 cells minimize carbon diversion to maximize fermentation rate and ethanol yield. The high-rate fermentation model in L1 allows the biofilm to maintain its primary industrial function while facing the highest levels of substrate availability. The transition to the L2 layer is marked by a significant “metabolic shift.” The peak accumulation of Glu and Pro—both downstream of the TCA cycle—signals a departure from the pure fermentation model toward a state of functional maturation. Furthermore, the elevated Met levels in L2, compared to L1, indicate a strategic diversion of carbon and sulfur resources toward the synthesis of precursors required for methylation and matrix reinforcement. In the deepest regions (B4I3 and B4I4), the observed decrease in amino acid accumulation compared to L2 suggests a tapering of structural investment. Instead of focusing on matrix reinforcement, L3 cells maintain a balanced metabolic pool to support the “protected niche” functions identified in our transcriptomic data—namely, ribosome biogenesis and cell cycle progression. By maintaining lower but stable metabolic diversion, L3 acts as a reproductive reservoir. These cells are shielded from external ethanol stress by the L1 and L2 barriers, preserving the population’s genetic and reproductive potential for biofilm self-renewal and “seeding” dispersal. This three-layer division of labor—fermentation (L1), scaffolding (L2), and proliferation (L3)—enables the biofilm to decouple immediate industrial performance from long-term population survival. The spatial partitioning of amino acid metabolism ensures that the immobilized system remains robust and productive over extended operational periods.

Our findings offer a toolkit for spatially aware synthetic biology. The identification of layer-specific markers (e.g., *CIT2* for outer layers, *FLO* genes for the scaffold, and *CLB6* for the core) enables the design of spatially inducible promoters. We envision “smart biofilms,” where heterologous pathways are compartmentalized: toxic catalytic steps could be restricted to the stress-tolerant outer layer (driven by RTG-responsive promoters), while pathway enzymes requiring labile cofactors could be localized to the protected inner core. This transition from “cell factory” to “structured community factory” represents a paradigm shift in industrial biotechnology.

In this study, we applied statistical approaches, treating mature biofilms with identical force and duration, and compared transcriptomes of cells eluted at different timepoints. Compared to FCF, BIF is more stable at the transcriptional level. In temporal dimension, the gene expression in FCF is centered around the survival of the cells. Unlike FCF, BIF showed no significant differences temporally, but, in spatial dimension, BIF achieved reproduction-metabolism decoupling: outer cells maximize glycolytic processes, middle cells maintain biofilm structure, and inner cells ensure division and reproduction.

### Limitations of the study

Several limitations of this study should be acknowledged. First, the spatial stratification of BCs was inferred using a sequential mechanical detachment strategy, in which differential adhesion strength was used as a proxy for biofilm depth. Although this approach enabled practical sampling from irregular cotton-fiber carriers under fermentation conditions, it does not provide direct *in situ* spatial resolution. Therefore, the defined L1-L3 layers should be interpreted as operationally separated subpopulations rather than sharply bounded anatomical layers. Second, the transcriptomic libraries were generated from pooled biological replicates, which reduced local stochastic variation but limited the ability to quantify replicate-level transcriptomic variability. Core findings were supported by quantitative reverse-transcription PCR (RT-qPCR), physiological measurements, and metabolite profiling; nevertheless, future studies using non-pooled RNA sequencing (RNA-seq), single-cell transcriptomics, spatial transcriptomics, or fluorescence-based *in situ* imaging would provide higher-resolution validation. Third, this work focused on one industrial *Saccharomyces cerevisiae* strain, one carrier material, and a specific biofilm-based immobilized fermentation configuration. The generality of the proposed reproduction-metabolism decoupling model across other yeast strains, carrier architectures, reactor scales, and fermentation products remains to be tested. Finally, several mechanistic interpretations, including retrograde signaling activation, carbon-flux redistribution, and layer-specific functional specialization, were mainly inferred from transcriptomic and metabolite patterns. Targeted genetic perturbation, isotope-assisted flux analysis, and direct measurements of local nutrient, oxygen, ethanol, and stress gradients will be necessary to establish causal relationships more rigorously.

## Resource availability

### Lead contact

Further information and requests for resources and reagents should be directed to and will be fulfilled by the lead contact, Yong Chen (chenyong1982@njtech.edu.cn).

### Materials availability

This study did not generate new unique reagents. An yeast strain (*Saccharomyces cerevisiae* 1308) generated in this study are available on request to the lead contact.

### Data and code availability

• The transcriptome sequencing data generated in this study have been deposited in the National Center for Biotechnology Information (NCBI) database under the BioProject accession number PRJNA1210668 (SRA: PRJNA1210668).
• This paper does not report original code.
• Any additional information required to reanalyze the data reported in this paper is available from the lead contact upon request.

## Acknowledgments

This work was supported by the 10.13039/501100012166National Key R&D Program of China (2025YFA0922300); the 10.13039/501100004608Natural Science Foundation of Jiangsu Province (BK20253004); the Jiangsu Basic Research Center for Synthetic Biology (BK20233003); the Jiangsu National Synergetic Innovation Center for Advanced Bio-Manufacture (XTA2201).

## Author contributions

C.L., writing – review and editing, writing – original draft, visualization, methodology, investigation, and data curation; J.R. and B.Y., writing – review and editing, and investigation; W.L., Q.L., and C.Z., resources, investigation, and writing – review and editing; J.S., S.S., and H.H., formal analysis, visualization, and data curation; W.S., writing – review and editing; Y.C., supervision, project administration, and funding acquisition.

## Declaration of interests

The authors declare no competing interests.

## STAR★Methods

### Key resources table

**Table:** 

REAGENT or RESOURCE	SOURCE	IDENTIFIER
**Chemicals, peptides, and recombinant proteins**		

Amino acids Mixture Standard (18)	Cadro	GB10261-1mL

**Deposited data**		

Transcriptomic Identifications	This study; SRA: PRJNA1210668	https://www.ncbi.nlm.nih.gov

**Software and algorithms**		

GraphPad Prism (version 10)	GraphPad Software	https://www.graphpad.com

### Experimental model and study participant details

This study did not involve experimental models or study participants, including animals, human participants, plants, microbe strains, cell lines, or primary cell cultures. No human-derived samples were used. Therefore, sex and gender were not considered.

### Method details

#### Strain and media

*S. cerevisiae* 1308,^18^ an ethanol-producing strain for industrial production grown on yeast extract peptone dextrose (YPD) agar plates (2% glucose, 2% peptone, 1% yeast extract, and 2% agar) at 30°C. The culture medium used for fermentation contained 50 g/L glucose, 4 g/L peptone, 4 g/L (NH_4_)_2_SO_4_, 3 g/L yeast extract, 3 g/L KH_2_PO_4_, 0.5 g/L MgSO_4_, 0.05 g/L ZnSO_4_·7H_2_O, and 0.05 g/L FeSO_4_·7H_2_O.^83^

#### Free-cell fermentation (FCF)

Pre-seed was cultured in 5 mL YPD medium in a 50 mL centrifuge tube at 30°C, 200 rpm for 12 h, and then transforming the pre-seed in a 250 mL shake flask containing 100 mL YPD medium culturing at 30°C, 200 rpm until the cell population density was 3 (OD_600_). The cultured samples were used as the seed for fermentation.

FCF started with 10 mL seed medium adding in 250 mL shake flasks containing 100 mL fermentation medium, and then the flasks were incubated at 35°C with shaking at 200 rpm. The fermentation ended with the residual glucose less than 1 g/L.

#### Biofilm-based immobilized fermentation (BIF)

The seed preparation process for BIF was the same as that for FCF. BIF fermentations started with 10 mL seed medium adding in 250 mL shake flasks containing 100 mL fermentation medium with 4 g carrier (a kind of cotton fiber providing for cell adhesion),^18^ and then the flasks were incubated at 35°C with shaking at 200 rpm. When the residual glucose less than 1 g/L, the fermented medium removed, leaving the cotton fiber carrier behind, and the fresh medium re-added into the flasks.

#### Analytical methods

Cell population density was determined by measuring optical density at 600 nm (OD_600_) using an ultraviolet spectrophotometer. Glucose concentrations in culture supernatants were quantified using the 3,5-dinitrosalicylic acid (DNS) colorimetric method. Ethanol production was analyzed by gas chromatography using an Agilent HP-INNOWAX column (60 m × 250 μm × 0.5 μm; Agilent Technologies, Santa Clara, CA, USA).^42^ Glycerol concentrations were determined by liquid chromatography (LC) analysis using an organic acid analysis column (BIO-RAD Aminex HPX-87H, 300 mm × 7.8 mm) following appropriate sample dilution after fermentation completion.

Trehalose^102^ and glycogen^103^ concentrations were measured upon fermentation termination. For biofilm-immobilized fermentation (BIF) samples, both planktonic cells and carrier-attached cells were collected. The carrier material was immersed in 100 mL of pre-cooled phosphate-buffered saline (PBS) and subjected to mechanical disruption using a vortex oscillator (Vibramax 100, 30W, Heidolph) for 10 s to detach BCs. The suspension was centrifuged, and the recovered cells were weighed. Cell disruption was performed using an ultrasonic cell disruptor (50% amplitude, 3-s pulses with 7-s intervals, total duration 50 min). Trehalose content was determined by LC analysis using a Waters Sugar-Pak1 column (Waters, USA) equipped with a refractive index detector, operating at a flow rate of 0.5 mL/min and column temperature of 80°C. Glycogen concentrations were quantified in duplicate using an enzymatic assay kit (EnzyChrom Glycogen Assay Kit, Biotrend, Cologne) according to the manufacturer’s protocol,^104^ with measurements performed on an Infinite M200 Tecan plate reader (Crailsheim, Germany). Both trehalose and glycogen concentrations were expressed as grams of metabolite per gram of dry cell weight (g ∗ g^−1^ DCW).

Budding population was determined by cell count technique.^19^ Samples were diluted with sterile water to 1 (OD_600_), then 50 μL methylene blue solution added to 10 mL samples and mix well. Place the clean coverslip over the hemocytometer, then use a dropper to aspirate and dispense several times to ensure the yeast cells are evenly distributed. Fill the wells of the hemocytometer with the thoroughly mixed dilution solution. The budding population was measured as the number of germinated cells dived by the total number of cells. Cell mortality was measured as the number of the stained cells dived by the total number of cells. Cell apoptosis was assessed using the TUNEL BrightRed Apoptosis Detection Kit (Vazyme, China) according to the manufacturer’s instructions, with quantitative analysis performed by flow cytometry in duplicate.

#### Flow cytometry

The cell cycle distribution of *S. cerevisiae* was determined by measuring cellular DNA content via flow cytometry, described by Liang et al.^42^ After harvesting, cells were collected by centrifugation (e.g., 5000 rpm, 5 min, 4°C) and the supernatant was discarded. The pellets were washed with ice-cold PBS and centrifuged again to ensure the removal of residual medium. For cell fixation and permeabilization, precooled 70% ethanol (at −20°C) was added slowly to resuspend the cells, followed by an overnight incubation in an ice bath. The fixed cells were then recovered by centrifugation (5000 rpm, 5 min, 4°C), washed with PBS, and resuspended in 250 μL of PBS. To eliminate interference from intracellular RNA, 12.5 μL of DNase-free RNase A (1 mg/L) was added to the suspension, which was then incubated in a water batch at 37°C for 40 min. After a subsequent wash with cold PBS, the cells were stained with Propidium Iodide (PI) at a final working concentration of 50 μg/mL in the dark for 20 min. To ensure accurate quantification of the DNA content in flocculent biofilm cells, the suspensions were subjected to repeated mechanical dissociation (pipetting) to achieve a single-cell state before analysis. Flow cytometric analysis was performed using a flow cytometer (BD Biosciences, Franklin Lakes, NJ, USA).

#### Analysis of intracellular amino acid content

A specific volume of fermentation broth (containing a known amount of biomass) was harvested and immediately centrifuged at 4,000 rpm for 5 min at 4°C. To remove extracellular amino acids and residual medium, the cell pellets were washed twice with ice-cold PBS (pH 7.4). The washed cells were then subjected to metabolic quenching and extraction. Then 5 mL of 80% (v/v) ethanol (precooled to −20 °C) were added to the cell pellets. The mixture was homogenized and the cells were disrupted using an ultrasonic cell crusher to release intracellular metabolites. The suspension was then incubated at 80 °C for 10 min and subsequently centrifuged at 12,000 rpm for 15 min at 4 °C. The supernatant containing the intracellular amino acids was collected and filtered through a 0.22 μm membrane for further analysis. The concentration of the extracted intracellular amino acids was determined using an automatic amino acid analyzer (S 7130; Sykam GmbH, Munich, Germany).

#### Sampling strategy and collection and transcriptomic analysis

For FCF, the planktonic cells were collected named FP1(SRR32001300), when the glucose concentration reaches three forth of the initial sugar, and the planktonic cells in the halved of glucose concentration named FP2 (SRR32001299). When the glucose concentration reached to one-fourth, planktonic cells named FP3 (SRR32001292) were collected, and the planktonic cells named FP4 (SRR32001291) were collected at the end of the fermentation.

For BIF, considering the complex geometry of the cotton fiber carrier, a sequential mechanical detachment method was employed to fractionate biofilm subpopulations based on their adhesion strength, which serves as a proxy for spatial distribution. Briefly, the carrier material containing biofilm (Figure S1) was immersed in 100 mL of pre-cooled PBS. The “outer layer” (L1) representing loosely attached cells was collected by an initial mechanical disruption using a vortex oscillator for 15 s. Subsequently, the “middle layer” (L2) and “inner layer” (L3) were obtained by repeating this process, assuming that inner cells require higher cumulative shear forces for detachment. This stratification allows for the investigation of spatial heterogeneity in gene expression despite the irregular carrier surface. Planktonic cells were harvested at 1-h intervals during the 8th batch cycle, yielding samples BP1 (SRR32001289), BP2 (SRR32001288), BP3 (SRR32001287), BP4 (SRR32001286), and BP5 (SRR32001285). BCs were collected via vortex oscillation (60 W, 15 s) at 2 h, 4 h and 5 h, designated B2I1 (SRR32001298), B4I1 (SRR32001297) and B5I1 (SRR32001296), respectively. Four samples of BCs were obtained from a carrier at 4 h using identical vortex parameters. The samples obtained for the first, second, third, and fourth times were labeled B4I1 (SRR32001297), B4I2 (SRR32001295), B4I3 (SRR32001294), and B4I4 (SRR32001293), respectively. Following collection, cells were immediately centrifuged (1,200 rpm, 4°C), washed three times with phosphate-buffered saline, snap-frozen in liquid nitrogen, and stored at −80°C. For easier reference and interpretation, all sample names and types listed in a summary figure in Figure S2 and a summary table in Table 1. All the samples were three replicates and were mixed in a 1:1:1 ratio.

Total RNA extraction, library construction, high-throughput sequencing, and bioinformatics analyses were conducted by Nanjing Anglai Biotechnology, Ltd. (Nanjing, China). Following quality control of raw sequencing data, reads were mapped to the *S. cerevisiae* reference genome GCF_000146045.2 (NCBI) to assess alignment efficiency and read distribution. Differentially expressed genes (DEGs) were identified using thresholds of |log_2_ fold change| ≥ 2.0 and false discovery rate (FDR)-adjusted *p* < 0.05. Functional enrichment analyses for Gene Ontology (GO) terms and Kyoto Encyclopedia of Genes and Genomes (KEGG) pathways were performed using the Database for Annotation, Visualization and Integrated Discovery (DAVID) v6.8 (https://davidbioinformatics.nih.gov) with stringency criteria of gene count ≥5 and EASE score ≤0.05.

#### Quantitative reverse transcription PCR (qRT-PCR)

Samples were collected as described in “sampling strategy and collection and transcriptomic analysis”, washed twice with phosphate-buffered saline (PBS), and ground in liquid nitrogen to disrupt the cell walls. Total RNA was then extracted using the MiniBEST Universal RNA Extraction Kit (TaKaRa, Kusatsu, Japan). Subsequently, cDNA libraries were synthesized using the HiScript II Q RT SuperMix for qPCR (+gDNA wiper; Vazyme, Nanjing, China). Gene-specific primers were designed based on reference sequences retrieved from the National Center for Biotechnology Information (NCBI) GenBank database (listed in Table S14). Quantitative real-time PCR (qRT-PCR) assays were performed using the AceQ qPCR SYBR Green Master Mix (Vazyme). Differentially expressed genes (DEGs) were identified using the EdgeR package with a screening threshold of a false discovery rate (FDR) < 0.05 and a ∣log_2_FC∣ > 1.^105^ All experiments were performed in triplicate.

### Quantification and statistical analysis

For transcriptomic analysis, total RNA was extracted from three independent biological replicates for each sample point. To obtain a representative profile of the biofilm community and minimize localized stochastic variations, these three RNA samples were pooled in an equal ratio (1:1:1) prior to library construction and sequencing. To ensure the robustness of the data, the core findings from the transcriptomic analysis were further validated by qRT-PCR and metabolic profiling, both of which were conducted using at least three independent biological replicates.

Data in Figures 1, 4F, 5F, 6G, and 6I were presented as mean ± standard derivated.

Samples excluding that used in transcriptome analysis were performed at least in triplicate, and data present the mean of three or more experiments. Differences between means were determined using the Student’s *t* test. Statistical significance was defined as *p* < 0.05 (∗), *p* < 0.01(∗∗), *p* < 0.001 (∗∗∗), and *p* < 0.0001 (∗∗∗∗). All statistical analyses were conducted using the built-in data fitting tools in Prism 10 software.

## Supplemental information

Document S1. Figures S1–S9

Table S1. Statistical table of identified upregulated and downregulated (DEGs)

Table S2. The main data of GO enrichment analysis in FCF in time dimension (blue: downregulated genes and red: upregulated genes)

Table S3. The main data of KEGG enrichment analysis in FCF in time dimension (blue: downregulated genes and red: upregulated genes)

Table S4. The main data of GO enrichment analysis of BPCs in time dimension (blue: downregulated genes and red: upregulated genes)

Table S5. The main data of KEGG enrichment analysis of BPCs in time dimension (blue: downregulated genes and red: upregulated genes)

Table S6. The main data of GO enrichment analysis of L1 in time dimension (blue: downregulated genes and red: upregulated genes)

Table S7. The main data of KEGG enrichment analysis of L1 in time dimension (blue: downregulated genes, red: upregulated genes, and black: non-significant variations)

Table S8. The main data of GO enrichment analysis of SVDs (blue: downregulated genes and red: upregulated genes)

Table S9. The main data of KEGG enrichment analysis of SVDs (blue: downregulated genes and red: upregulated genes)

Table S10. The main data of GO enrichment analysis of DEGs shared between BPCs and L1 (blue: downregulated genes and red: upregulated genes)

Table S11. The main data of KEGG enrichment analysis of DEGs shared between BPCs and L1 (blue: downregulated genes and red: upregulated genes)

Table S12. The main data of GO enrichment analysis in biofilm cells in the spatial dimension (blue: downregulated genes and red: upregulated genes)

Table S13. The main data of KEGG enrichment analysis in biofilm cells in the spatial dimension (blue: downregulated genes and red: upregulated genes)

Table S14. The primer sequence utilized for RT-qPCR

## References

[1] Costerton J. (1995). Overview of microbial biofilms. J. Ind. Microbiol..

[2] Costerton J.W., Lewandowski Z. (1997). The Biofilm Lifestyle. Adv. Dent. Res..

[3] Jiang Y., Liu Y., Zhang X., Gao H., Mou L., Wu M., Zhang W., Xin F., Jiang M. (2021). Biofilm application in the microbial biochemicals production process. Biotechnol. Adv..

[4] Alvarez-Ordóñez A., Coughlan L.M., Briandet R., Cotter P.D. (2019). Biofilms in Food Processing Environments: Challenges and Opportunities. Annu. Rev. Food Sci. Technol..

[5] Lee Wong A.C. (1998). Biofilms in Food Processing Environments. J. Dairy Sci..

[6] Zhao X., Zhao F., Wang J., Zhong N. (2017). Biofilm formation and control strategies of foodborne pathogens: food safety perspectives. RSC Adv..

[7] Costerton J.W., Montanaro L., Arciola C.R. (2005). Biofilm in implant infections: Its production and regulation. Int. J. Artif. Organs.

[8] Fang K., Park O.J., Hong S.H. (2020). Controlling biofilms using synthetic biology approaches. Biotechnol. Adv..

[9] Yang L., Zheng C., Chen Y., Ying H. (2018). *FLO* Genes Family and Transcription Factor *MIG1* Regulate *Saccharomyces cerevisiae* Biofilm Formation During Immobilized Fermentation. Front. Microbiol..

[10] Roostaei J., Zhang Y., Gopalakrishnan K., Ochocki A.J. (2018). Mixotrophic Microalgae Biofilm: A Novel Algae Cultivation Strategy for Improved Productivity and Cost-efficiency of Biofuel Feedstock Production. Sci. Rep..

[11] Lieleg O., Caldara M., Baumgärtel R., Ribbeck K. (2011). Mechanical robustness of *Pseudomonas aeruginosa* biofilms. Soft Matter.

[12] Wang Y.J., Liao Q., Wang Y.Z., Zhu X., Li J. (2011). Effects of flow rate and substrate concentration on the formation and H2 production of photosynthetic bacterial biofilms. Bioresour. Technol..

[13] Carvalho F.M., Azevedo A., Ferreira M.M., Mergulhão F.J.M., Gomes L.C. (2022). Advances on Bacterial and Fungal Biofilms for the Production of Added-Value Compounds. Biology.

[14] Liang C., Wang Y., Shi S., Yan A., Liu Q., Liu W., Han H., Qi W., Chen T., Sun W., Chen Y. (2025). Integrating Biofilm-Based Fermentation With Continuous Processes for Improved L-Valine Production. Biotechnol. J..

[15] Han H., Zhang K., Li G., Yu Y., Shi S., Liang C., Niu H., Zhuang W., Liu D., Yang P. (2023). Autoinducer-2: Its Role in Biofilm Formation and L-Threonine Production in *Escherichia coli*. Fermentation.

[16] Liu D., Chen Y., Ding F.-Y., Zhao T., Wu J.-L., Guo T., Ren H.-F., Li B.-B., Niu H.-Q., Cao Z. (2014). Biobutanol production in a *Clostridium acetobutylicum* biofilm reactor integrated with simultaneous product recovery by adsorption. Biotechnol. Biofuels.

[17] Qureshi N., Schripsema J., Lienhardt J., Blaschek H.P. (2000). Continuous solvent production by *Clostridium beijerinckii* BA101 immobilized by adsorption onto brick. World J. Microb. Biotechnol..

[18] Li Z., Chen Y., Liu D., Zhao N., Cheng H., Ren H., Guo T., Niu H., Zhuang W., Wu J., Ying H. (2015). Involvement of glycolysis/gluconeogenesis and signaling regulatory pathways in *Saccharomyces cerevisiae* biofilms during fermentation. Front. Microbiol..

[19] Liu Q., Zhao N., Zou Y., Ying H., Chen Y. (2020). Feasibility of ethanol production from expired rice by surface immobilization technology in a new type of packed bed pilot reactor. Renew. Energy.

[20] Chen T., Liu N., Ren P., Xi X., Yang L., Sun W., Yu B., Ying H., Ouyang P., Liu D., Chen Y. (2019). Efficient Biofilm-Based Fermentation Strategies for L-Threonine Production by *Escherichia coli*. Front. Microbiol..

[21] Yao S., Hao L., Zhou R., Jin Y., Huang J., Wu C. (2022). Multispecies biofilms in fermentation: Biofilm formation, microbial interactions, and communication. Compr. Rev. Food Sci. Food Saf..

[22] Čáp M., Váchová L., Palková Z. (2015). Longevity of U cells of differentiated yeast colonies grown on respiratory medium depends on active glycolysis. Cell Cycle.

[23] Plocek V., Fadrhonc K., Maršíková J., Váchová L., Pokorná A., Hlaváček O., Wilkinson D., Palková Z. (2021). Mitochondrial Retrograde Signaling Contributes to Metabolic Differentiation in Yeast Colonies. Int. J. Mol. Sci..

[24] Čáp M., Štěpánek L., Harant K., Váchová L., Palková Z. (2012). Cell differentiation within a yeast colony: metabolic and regulatory parallels with a tumor-affected organism. Mol. Cell.

[25] Gordan J.D., Thompson C.B., Simon M.C. (2007). HIF and c-Myc: sibling rivals for control of cancer cell metabolism and proliferation. Cancer Cell.

[26] Chao S., Woolford J.L. (1994). The yeast *NOP4* gene product is an essential nucleolar protein required for pre-rRNA processing and accumulation of 60S ribosomal subunits. EMBO J..

[27] Li S., Xu X., Zhao T., Ma J., Zhao L., Song Q., Sun W. (2022). Screening of Bacillus velezensis E2 and the Inhibitory Effect of Its Antifungal Substances on *Aspergillus flavus*. Foods.

[28] Takaine M., Morita R., Yoshinari Y., Nishimura T. (2025). Phase separation of the PRPP amidotransferase into dynamic condensates promotes de novo purine synthesis in yeast. PLoS Biol..

[29] Greatrix B.W., van Vuuren H.J.J. (2006). Expression of the *HXT13, HXT15* and *HXT17* genes in *Saccharomyces cerevisiae* and stabilization of the *HXT1* gene transcript by sugar-induced osmotic stress. Curr. Genet..

[30] Zhang P., Du G., Zou H., Chen J., Xie G., Shi Z., Zhou J. (2016). Effects of three permeases on arginine utilization in *Saccharomyces cerevisiae*. Sci. Rep..

[31] Manzoor Y., Aouida M., Ramadoss R., Moovarkumudalvan B., Ahmed N., Sulaiman A.A., Mohanty A., Ali R., Mifsud B., Ramotar D. (2024). Loss of the yeast transporter Agp2 upregulates the pleiotropic drug-resistant pump Pdr5 and confers resistance to the protein synthesis inhibitor cycloheximide. PLoS One.

[32] Shaw K.J. (2022). GR-2397: Review of the Novel Siderophore-like Antifungal Agent for the Treatment of Invasive Aspergillosis. J. Fungi.

[33] Ko C.H., Gaber R.F. (1991). *TRK1* and *TRK2* Encode Structurally Related K^+^ Transporters in *Saccharomyces cerevisiae*. Mol. Cell Biol..

[34] Nozawa A., Takano J., Kobayashi M., von Wirén N., Fujiwara T. (2006). Roles of BOR1, DUR3, and FPS1 in boron transport and tolerance in *Saccharomyces cerevisiae*. FEMS Microbiol. Lett..

[35] Ishii R., Fukui A., Sakihama Y., Kitsukawa S., Futami A., Mochizuki T., Nagano M., Toshima J., Abe F. (2022). Substrate-induced differential degradation and partitioning of the two tryptophan permeases Tat1 and Tat2 into eisosomes in *Saccharomyces cerevisiae*. Biochim. Biophys. Acta Biomembr..

[36] Nishimura A. (2024). Regulations and functions of proline utilization in yeast *Saccharomyces cerevisiae*. Biosci. Biotechnol. Biochem..

[37] Zimmermannova O., Felcmanova K., Sacka L., Colinet A.S., Morsomme P., Sychrova H. (2021). K^+^-specific importers Trk1 and Trk2 play different roles in Ca^2+^ homeostasis and signalling in *Saccharomyces cerevisiae* cells. FEMS Yeast Res..

[38] Wang L., Li B., Su R.R., Wang S.P., Xia Z.Y., Xie C.Y., Tang Y.Q. (2022). Screening novel genes by a comprehensive strategy to construct multiple stress-tolerant industrial *Saccharomyces cerevisiae* with prominent bioethanol production. Biotechnol. Biofuels Bioprod..

[39] Velasco I., Tenreiro S., Calderon I.L., André B. (2004). *Saccharomyces cerevisiae* Aqr1 is an internal-membrane transporter involved in excretion of amino acids. Eukaryot. Cell.

[40] Paumi C.M., Chuk M., Snider J., Stagljar I., Michaelis S. (2009). ABC transporters in *Saccharomyces cerevisiae* and their interactors: new technology advances the biology of the ABCC (MRP) subfamily. Microbiol. Mol. Biol. Rev..

[41] Donati G., Montanaro L., Derenzini M. (2012). Ribosome biogenesis and control of cell proliferation: p53 is not alone. Cancer Res..

[42] Liang C., Ding S., Sun W., Liu L., Zhao W., Zhang D., Ying H., Liu D., Chen Y. (2020). Biofilm-based fermentation: a novel immobilisation strategy for *Saccharomyces cerevisiae* cell cycle progression during ethanol production. Appl. Microbiol. Biotechnol..

[43] Talarek N., Gueydon E., Schwob E. (2017). Homeostatic control of START through negative feedback between Cln3-Cdk1 and Rim15/Greatwall kinase in budding yeast. eLife.

[44] Kozak B.U., van Rossum H.M., Niemeijer M.S., van Dijk M., Benjamin K., Wu L., Daran J.M.G., Pronk J.T., van Maris A.J.A. (2016). Replacement of the initial steps of ethanol metabolism in *Saccharomyces cerevisiae* by ATP-independent acetylating acetaldehyde dehydrogenase. FEMS Yeast Res..

[45] Tennyson C.N., Lee J., Andrews B.J. (1998). A role for the Pcl9-Pho85 cyclin-cdk complex at the M/G1 boundary in *Saccharomyces cerevisiae*. Mol. Microbiol..

[46] Yu S., Meng S., Xiang M., Ma H. (2021). Phosphoenolpyruvate carboxykinase in cell metabolism: Roles and mechanisms beyond gluconeogenesis. Mol. Metab..

[47] Nagarajan S., Kruckeberg A.L., Schmidt K.H., Kroll E., Hamilton M., McInnerney K., Summers R., Taylor T., Rosenzweig F. (2014). Uncoupling reproduction from metabolism extends chronological lifespan in yeast. Proc. Natl. Acad. Sci. USA.

[48] Ainscow E.K., Brand M.D. (1999). Top-down control analysis of ATP turnover, glycolysis and oxidative phosphorylation in rat hepatocytes. Eur. J. Biochem..

[49] Larsson C., Påhlman I., Gustafsson L. (2000). The importance of ATP as a regulator of glycolytic flux in *Saccharomyces cerevisiae*. Yeast.

[50] Brosnan J.T., Brosnan M.E. (2006). The sulfur-containing amino acids: an overview. J. Nutr..

[51] Pérez D., Jaehde I., Guillamón J.M., Heras J.M., Querol A. (2021). Screening of *Saccharomyces* strains for the capacity to produce desirable fermentative compounds under the influence of different nitrogen sources in synthetic wine fermentations. Food Microbiol..

[52] Takagi H. (2019). Metabolic regulatory mechanisms and physiological roles of functional amino acids and their applications in yeast. Biosci. Biotechnol. Biochem..

[53] Thomas K.C., Ingledew W.M. (1992). Relationship of low lysine and high arginine concentration to efficient ethanolic fermentation of wheat mash. Can. J. Microbiol..

[54] Chen X., Nielsen K.F., Borodina I., Kielland-Brandt M.C., Karhumaa K. (2011). Increased isobutanol production in *Saccharomyces cerevisiae* by overexpression of genes in valine metabolism. Biotechnol. Biofuels.

[55] Chrzanowski G. (2020). *Saccharomyces Cerevisiae*-An Interesting Producer of Bioactive Plant Polyphenolic Metabolites. Int. J. Mol. Sci..

[56] Sonawala U., Busidan A., Haak D., Pilot G. (2025). Characterization and whole genome sequencing of *Saccharomyces cerevisiae* strains lacking several amino acid transporters: Tools for studying amino acid transport. PLoS One.

[57] Nakajima T., Hosoyamada S., Kobayashi T., Mukai Y. (2022). Secreted acid phosphatases maintain replicative lifespan via inositol polyphosphate metabolism in budding yeast. FEBS Lett..

[58] Mouillon J.M., Persson B.L. (2006). New aspects on phosphate sensing and signalling in *Saccharomyces cerevisiae*. FEMS Yeast Res..

[59] Dong X.Y. (2023). Calcium Ion Channels in *Saccharomyces cerevisiae*. J. Fungi.

[60] Ma T.Y., Deprez M.A., Callewaert G., Winderickx J. (2021). Coordinated glucose-induced Ca^2+^ and pH responses in yeast *Saccharomyces cerevisiae*. Cell Calcium.

[61] Sabir F., Loureiro-Dias M.C., Soveral G., Prista C. (2017). Functional relevance of water and glycerol channels in *Saccharomyces cerevisiae*. FEMS Microbiol. Lett..

[62] Yang Y., Xie P., Li Y., Bi Y., Prusky D.B. (2022). Updating Insights into the Regulatory Mechanisms of Calcineurin-Activated Transcription Factor Crz1 in Pathogenic Fungi. J. Fungi.

[63] Zekhnini A., Albacar M., Casamayor A., Ariño J. (2023). The ENA1 Na^+^-ATPase Gene Is Regulated by the SPS Sensing Pathway and the Stp1/Stp2 Transcription Factors. Int. J. Mol. Sci..

[64] Shirvanyan A., Trchounian K. (2024). Sodium transport and redox regulation in *Saccharomyces cerevisiae* under osmotic stress depending on oxygen availability. Sci. Rep..

[65] Zahrádka J., Sychrová H. (2012). Plasma-membrane hyperpolarization diminishes the cation efflux via Nha1 antiporter and Ena ATPase under potassium-limiting conditions. FEMS Yeast Res..

[66] Pan H.P., Wang N., Tachikawa H., Gao X.D., Nakanishi H. (2018). Osw2 is required for proper assembly of glucan and/or mannan layers of the yeast spore wall. J. Biochem..

[67] Krause D.J., Hittinger C.T. (2022). Functional Divergence in a Multi-gene Family Is a Key Evolutionary Innovation for Anaerobic Growth in *Saccharomyces cerevisiae*. Mol. Biol. Evol..

[68] Nasution O., Lee J., Srinivasa K., Choi I.G., Lee Y.M., Kim E., Choi W., Kim W. (2015). Loss of Dfg5 glycosylphosphatidylinositol-anchored membrane protein confers enhanced heat tolerance in *Saccharomyces cerevisiae*. Environ. Microbiol..

[69] Nishimura A., Tanahashi R., Nakagami K., Morioka Y., Takagi H. (2024). The arginine transporter Can1 negatively regulates biofilm formation in yeasts. Front. Microbiol..

[70] Tanahashi R., Nishimura A., Morita F., Nakazawa H., Taniguchi A., Ichikawa K., Nakagami K., Boundy-Mills K., Takagi H. (2023). The arginine transporter Can1 acts as a transceptor for regulation of proline utilization in the yeast *Saccharomyces cerevisiae*. Yeast.

[71] Tanaka N., Mukai Y. (2015). Yeast Cyc8p and Tup1p proteins function as coactivators for transcription of Stp1/2p-dependent amino acid transporter genes. Biochem. Biophys. Res. Commun..

[72] Jiang S., Li H., Piao L., Jin Z., Liu J., Chen S., Liu L.L., Shao Y., Zhong S., Wu B. (2020). Computational study on new natural compound inhibitors of indoleamine 2,3-dioxygenase 1. Aging.

[73] Takano J., Kobayashi M., Noda Y., Fujiwara T. (2007). *Saccharomyces cerevisiae* Bor1p is a boron exporter and a key determinant of boron tolerance. FEMS Microbiol. Lett..

[74] Zhang Y., Ma Z., Li W., Liu C., Gao H., Wang M., Li L., Zhang Q., Lv B., Qin L., Li C. (2025). Dynamic regulation and enhancement of synthetic network for efficient biosynthesis of monoterpenoid alpha-pinene in yeast cell factory. Bioresour. Technol..

[75] AE E.-M., FE W., C S. (1994). The *saccharomyces cerevisiae SGE1* gene product: a novel drug-resistance protein within the major facilitator superfamily. Mol. Gen. Genet..

[76] Verce M., Schoonejans J., Hernandez Aguirre C., Molina-Bravo R., De Vuyst L., Weckx S. (2021). A Combined Metagenomics and Metatranscriptomics Approach to Unravel Costa Rican Cocoa Box Fermentation Processes Reveals Yet Unreported Microbial Species and Functionalities. Front. Microbiol..

[77] Law D.T., Segall J. (1988). The *SPS100* Gene of *Saccharomyces cerevisiae* Is Activated Late in the Sporulation Process and Contributes to Spore Wall Maturation. Mol. Cell Biol..

[78] de Souza C.M., Paulo E.A., de Souza N.A.A., Dos Santos M.M., Mantovani M.S., Furlaneto-Maia L., Furlaneto M.C. (2025). Enhanced biofilm formation by *Candida tropicalis* morphotypes under host-mimicking conditions: Insights into cell wall modifications and gene expression. Microb. Pathog..

[79] Kasavi C., Eraslan S., Oner E.T., Kirdar B. (2015). An integrative analysis of transcriptomic response of ethanol tolerant strains to ethanol in *Saccharomyces cerevisiae*. Mol. Biosyst..

[80] Soares E.V. (2011). Flocculation in *Saccharomyces cerevisiae*: a review. J. Appl. Microbiol..

[81] Stolz J., Sauer N. (1999). The Fenpropimorph Resistance Gene *FEN2* from *Saccharomyces cerevisiae* Encodes a Plasma Membrane H^+^-Pantothenate Symporter. J. Biol. Chem..

[82] Marcireau C., Joets J., Pousset D., Guilloton M., Karst F. (1996). *FEN2*: A Gene Implicated in the Catabolite Repression-Mediated Regulation of Ergosterol Biosynthesis in Yeast. Yeast.

[83] Yang L., Zheng C., Chen Y., Shi X., Ying Z., Ying H. (2019). Nitric oxide increases biofilm formation in *Saccharomyces cerevisiae* by activating the transcriptional factor Mac1p and thereby regulating the transmembrane protein Ctr1. Biotechnol. Biofuels.

[84] Petkova M.I., Pujol-Carrion N., de la Torre-Ruiz M.A. (2012). Mtl1 O-mannosylation mediated by both Pmt1 and Pmt2 is important for cell survival under oxidative conditions and TOR blockade. Fungal Genet. Biol..

[85] Hau Lam Choy E.A.G., Doering T.L. (2023). Ergosterol distribution controls surface structure formaton and fungal pathogenicity. mBio.

[86] Bui T.H.D., Labedzka-Dmoch K. (2024). RetroGREAT signaling: The lessons we learn from yeast. IUBMB Life.

[87] Rios-Anjos R.M., Camandona V.d.L., Bleicher L., Ferreira-Junior J.R. (2017). Structural and functional mapping of Rtg2p determinants involved in retrograde signaling and aging of *Saccharomyces cerevisiae*. PLoS One.

[88] Liu Z., Butow R.A. (2006). Mitochondrial retrograde signaling. Annu. Rev. Genet..

[89] Trendeleva T.A., Zvyagilskaya R.A. (2018). Retrograde Signaling as a Mechanism of Yeast Adaptation to Unfavorable Factors. Biochemistry (Moscow).

[90] Sekito T., Thornton J., Butow R.A. (2000). Mitochondria-to-nuclear signaling is regulated by the subcellular localization of the transcription factors Rtg1p and Rtg3p. Mol. Biol. Cell.

[91] Meneèndez j., Gancedo C. (1998). Regulatory regions in the promoters of the *saccharomyces cerevisiae PYC1* and *PYC2* genes encoding isoenzymes of pyruvate carboxylase. FEMS Microbiol. Lett..

[92] Lorenz M.C., Fink G.R. (2001). The glyoxylate cycle is required for fungal virulence. Nature.

[93] Giannattasio S., Liu Z., Thornton J., Butow R.A. (2005). Retrograde response to mitochondrial dysfunction is separable from TOR1/2 regulation of retrograde gene expression. J. Biol. Chem..

[94] Zhang W., Du G., Zhou J., Chen J. (2018). Regulation of Sensing, Transportation, and Catabolism of Nitrogen Sources in *Saccharomyces cerevisiae*. Microbiol. Mol. Biol. Rev..

[95] Moradas-Ferreira P., Costa V. (2000). Adaptive response of the yeast *Saccharomyces cerevisiae* to reactive oxygen species: defences, damage and death. Redox Rep..

[96] Yang P., Jiang S., Jiang S., Lu S., Zheng Z., Chen J., Wu W., Jiang S. (2022). CRISPR-Cas9 Approach Constructed Engineered *Saccharomyces cerevisiae* with the Deletion of *GPD2, FPS1*, and *ADH2* to Enhance the Production of Ethanol. J. Fungi.

[97] Zara G., Bou Zeidan M., Fancello F., Sanna M.L., Mannazzu I., Budroni M., Zara S. (2019). The administration of L-cysteine and L-arginine inhibits biofilm formation in wild-type biofilm-forming yeast by modulating *FLO11* gene expression. Appl. Microbiol. Biotechnol..

[98] Shrivastava M., Feng J., Coles M., Clark B., Islam A., Dumeaux V., Whiteway M. (2021). Modulation of the complex regulatory network for methionine biosynthesis in fungi. Genetics.

[99] Jochim A., Shi T., Belikova D., Schwarz S., Peschel A., Heilbronner S. (2019). Methionine Limitation Impairs Pathogen Expansion and Biofilm Formation Capacity. Appl. Environ. Microbiol..

[100] Dal Co A., Brenner M.P. (2020). Tracing cell trajectories in a biofilm. Science.

[101] Jiang Y., Liang C., Zhao W., Chen T., Yu B., Hou A., Zhu J., Zhang T., Liu Q., Ying H. (2022). Cell cycle progression influences biofilm formation in *saccharomyces cerevisiae* 1308. Microbiol. Spectr..

[102] Yi C., Wang F., Dong S., Li H. (2016). Changes of trehalose content and expression of relative genes during the bioethanol fermentation by *Saccharomyces cerevisiae*. Can. J. Microbiol..

[103] Primassin S., Tucci S., Spiekerkoetter U. (2011). Hepatic and muscular effects of different dietary fat content in VLCAD deficient mice. Mol. Genet. Metab..

[104] Hunter A.L., Pelekanou C.E., Adamson A., Downton P., Barron N.J., Cornfield T., Poolman T.M., Humphreys N., Cunningham P.S., Hodson L. (2020). Nuclear receptor REVERBalpha is a state-dependent regulator of liver energy metabolism. Proc. Natl. Acad. Sci. USA.

[105] Sun W., Liu L., Yu Y., Yu B., Liang C., Ying H., Liu D., Chen Y. (2020). Biofilm-Related, Time-Series Transcriptome and Genome Sequencing in Xylanase-Producing *Aspergillus niger* SJ1. ACS Omega.

