# Supplementary material for: Transcriptomics reveals metabolic division of labor and reproduction-metabolism decoupling in Saccharomyces cerevisiae biofilms
Source: iScience. 2026 Sep 9;29(9):117407. doi: 10.1016/j.isci.2026.117407 (PMC13582009; doi:10.1016/j.isci.2026.117407)
Supplement: Supplementary file 1 [file mmc1.pdf]

iScience, Volume *29*

Supplemental information

Transcriptomics reveals metabolic division of labor and reproduction-metabolism decoupling in *Saccharomyces cerevisiae* biofilms

Caice Liang, Jing Ruan, Biao Yang, Wei Liu, Qingguo Liu, Chaowei Zhou, Junqiang Shan, Shuqi Shi, Hui Han, Wenjun Sun, and Yong Chen

Figures

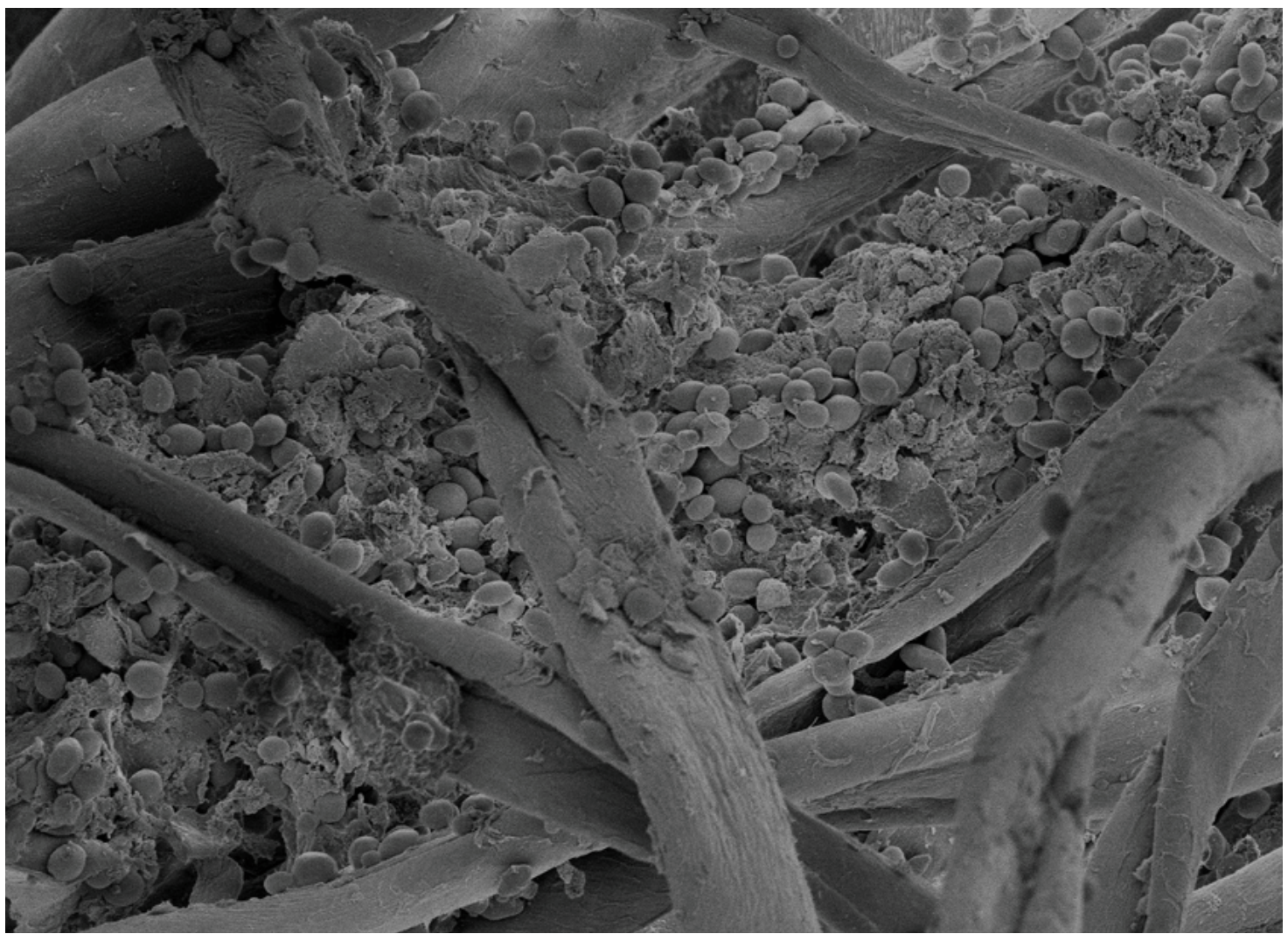

Figure S1 The carrier material contains biofilm.

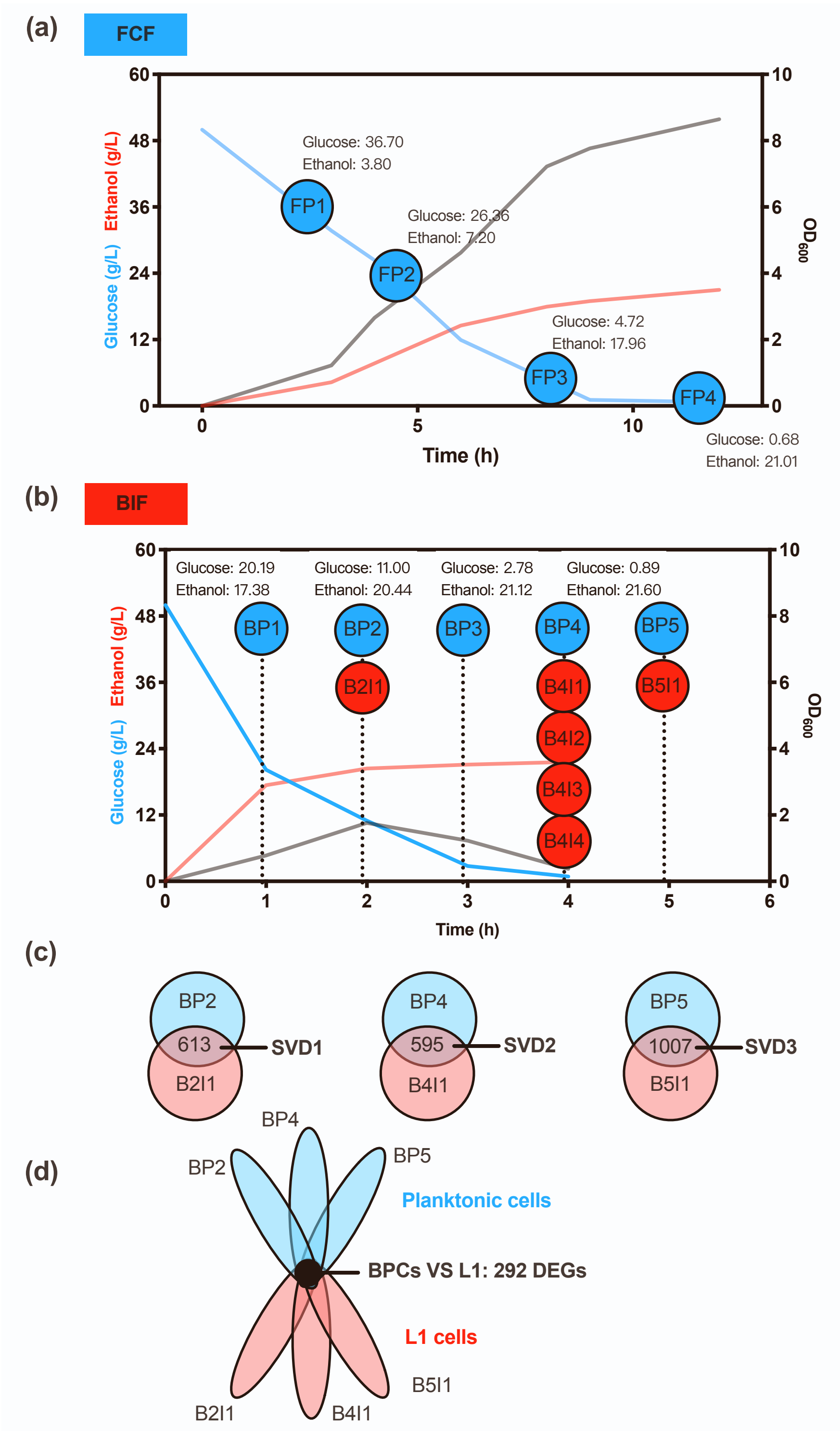

Fig. S2 Summary of sample nomenclature and classification. (a) Blue circles represent planktonic cells from FCF (FP1, FP2, FP3, and FP4). (b) Blue circles represent planktonic cells from BIF (BP1, BP2, BP3, and BP4). Red circles represent biofilm cells collected from the carriers. (c) SVG1, SVG2, and SVG3 represent the shared DEGs between BP2 and B2I2, BP4 and B4I1, and BP5 and B5I1. (d) BPCs vs L1 were the shared DEGs in planktonic cells and L1.

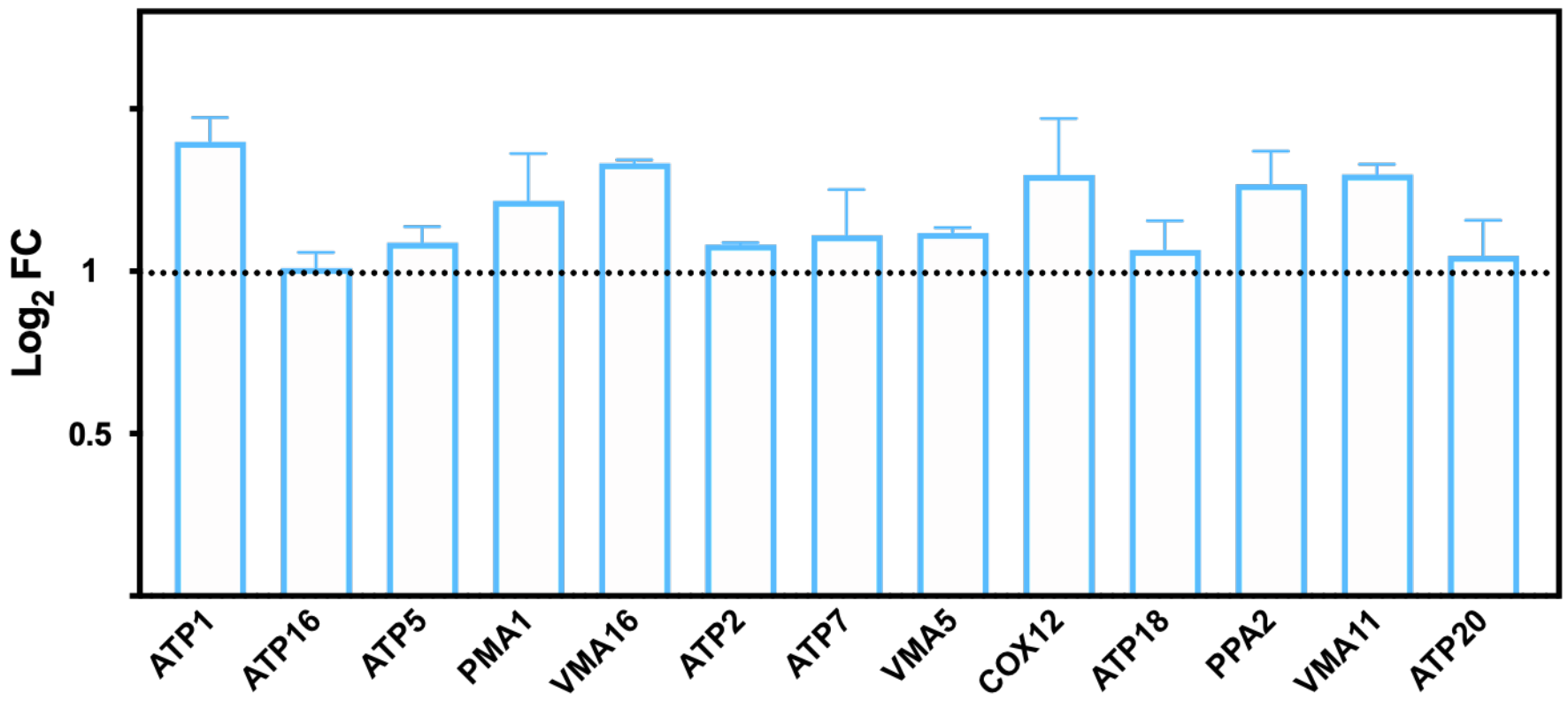

Fig. S3 Difference in gene expression related to ATP synthesis of FCs in BIF in SVD3. Data are represented as mean ± SEM (n = 3 biological replicates per group).

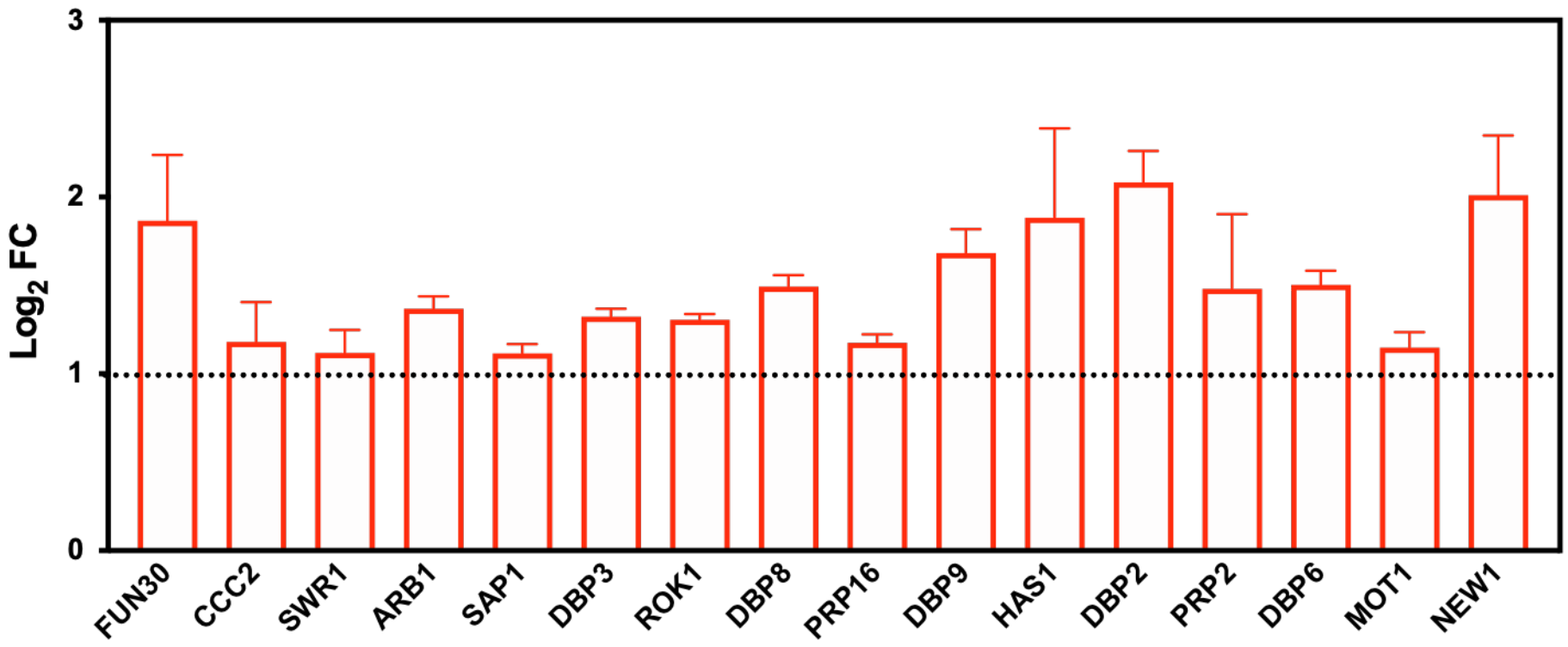

Fig. S4 Data are represented as mean ± standard deviation. Difference in gene expression related to ATP utilization of L1 in SVD3. Data are represented as mean ± SEM (n = 3 biological replicates per group).

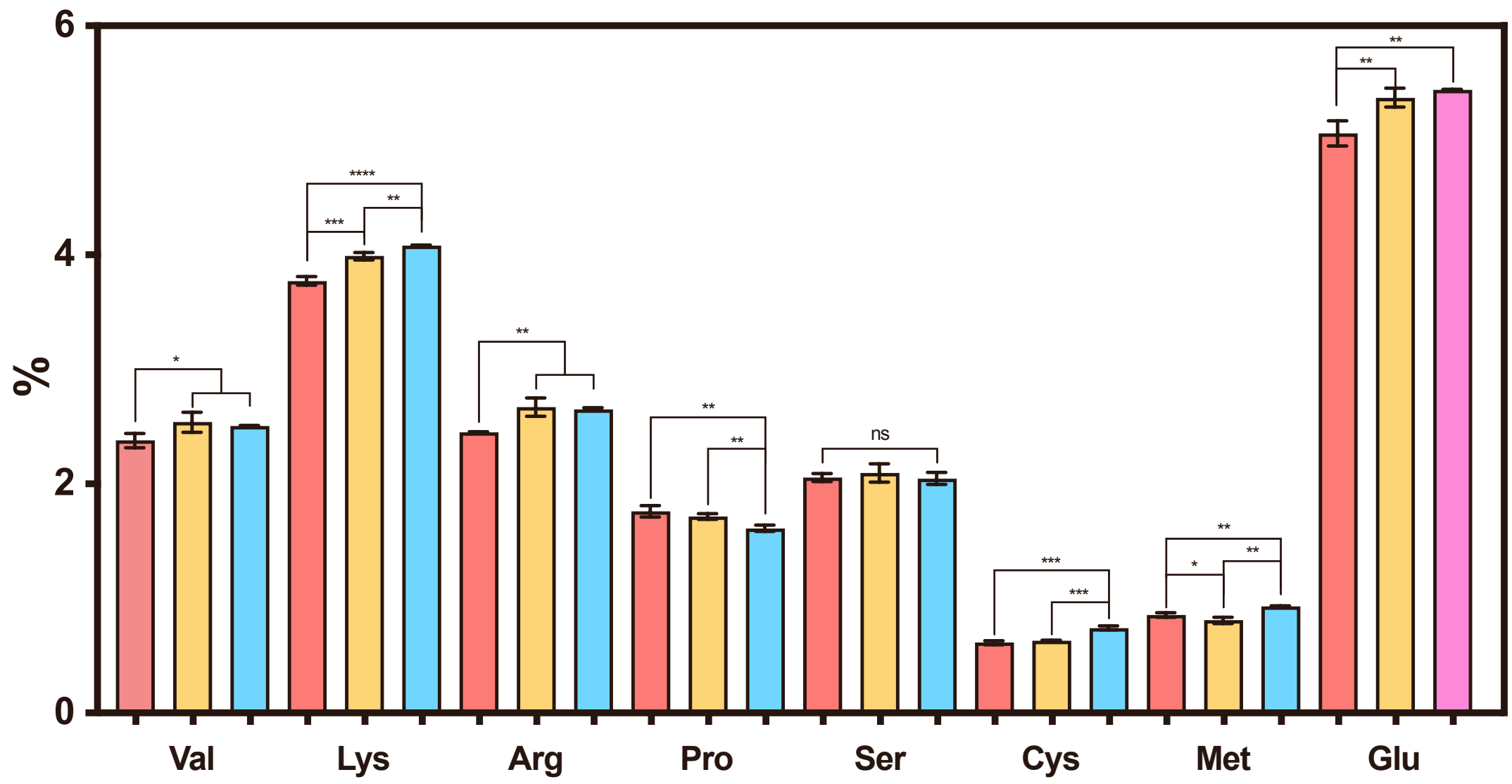

Fig. S5 Data are represented as mean ± standard deviation. The levels of amino acids of BPCs (Red represented amino acid levels in BP2, yellow represented amino acid levels in BP4, and blue represented amino acid levels in BP5). Data are represented as mean ± SEM; ns $p > 0.05$, *$p < 0.05$, **$p < 0.01$, ***$p < 0.001$, ****$p < 0.0001$ by one-way ANOVA (n = 3 biological replicates per group).

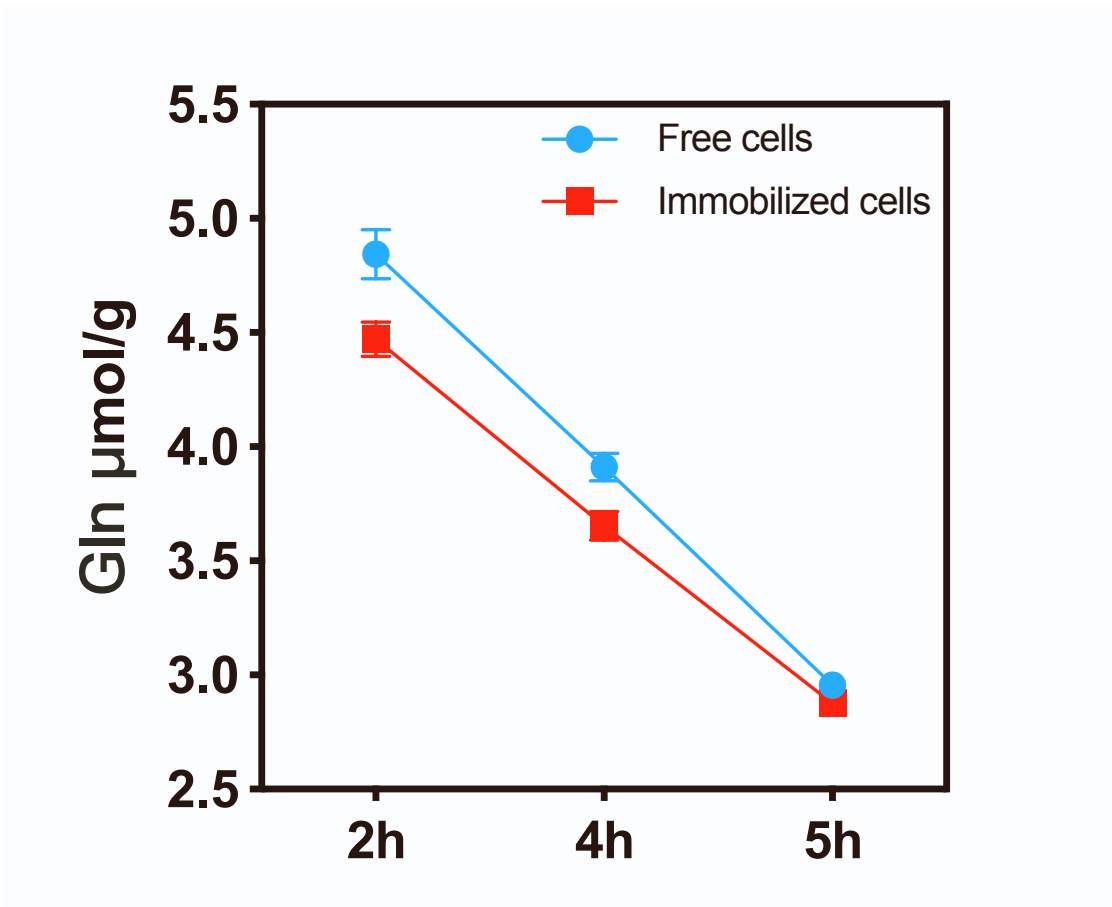

Fig. S6 The levels of Glutamine (Gln) of free cells (blue cycles) and immobilized cells (red squares) in biofilms. Data are represented as mean ± SEM; ns $p > 0.05$, *$p < 0.05$, **$p < 0.01$, ***$p < 0.001$, ****$p < 0.0001$ by one-way ANOVA (n = 3 biological replicates per group).

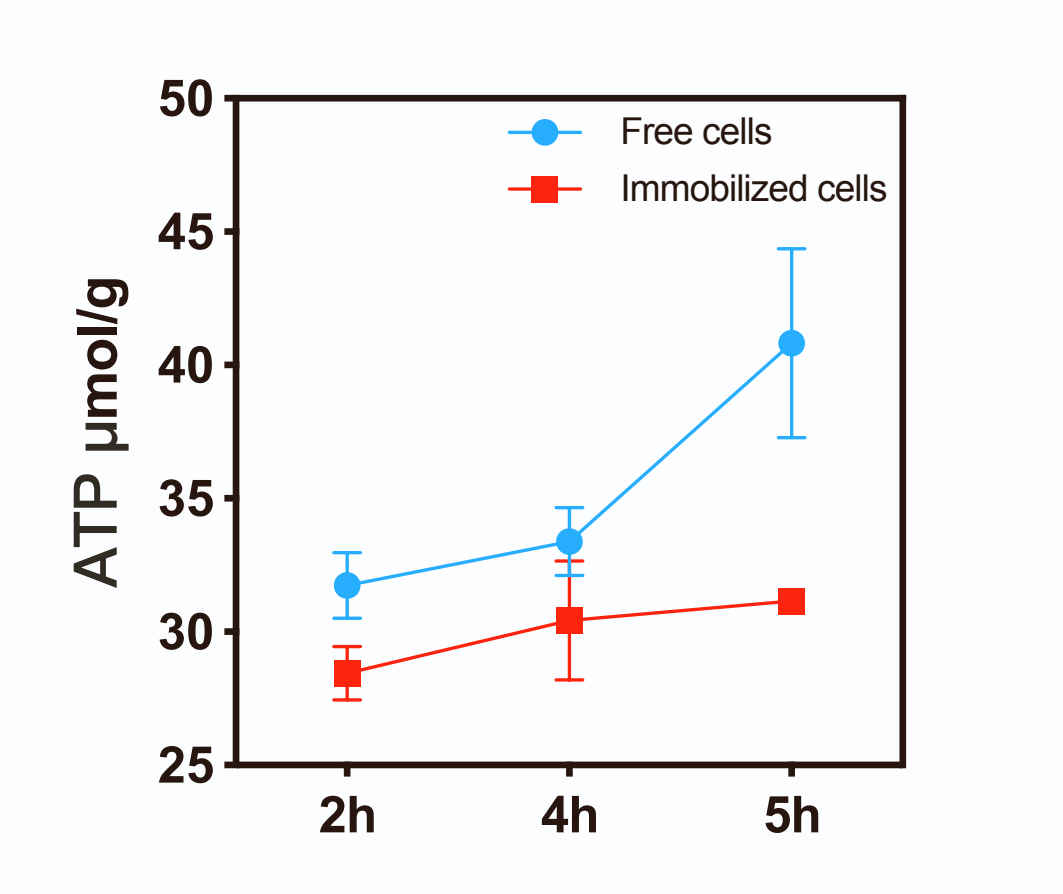

Fig. S7 The levels of ATP of free cells (blue cycles) and immobilized cells (red squares) in biofilms. Data are represented as mean ± SEM; ns $p > 0.05$, *$p < 0.05$, **$p < 0.01$, ***$p < 0.001$, ****$p < 0.0001$ by one-way ANOVA (n = 3 biological replicates per group).

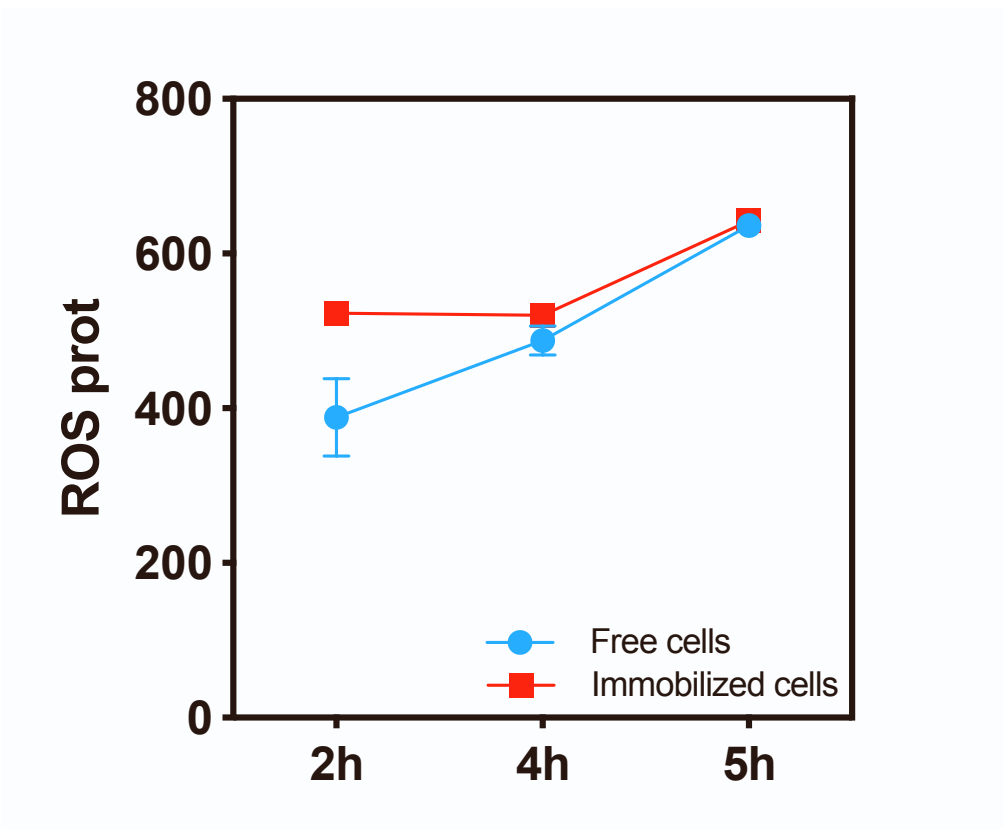

Fig. S8 The levels of ROS of free cells (blue cycles) and immobilized cells (red squares) in biofilms. Data are represented as mean ± SEM; ns $p > 0.05$, *$p < 0.05$, **$p < 0.01$, ***$p < 0.001$, ****$p < 0.0001$ by one-way ANOVA (n = 3 biological replicates per group).

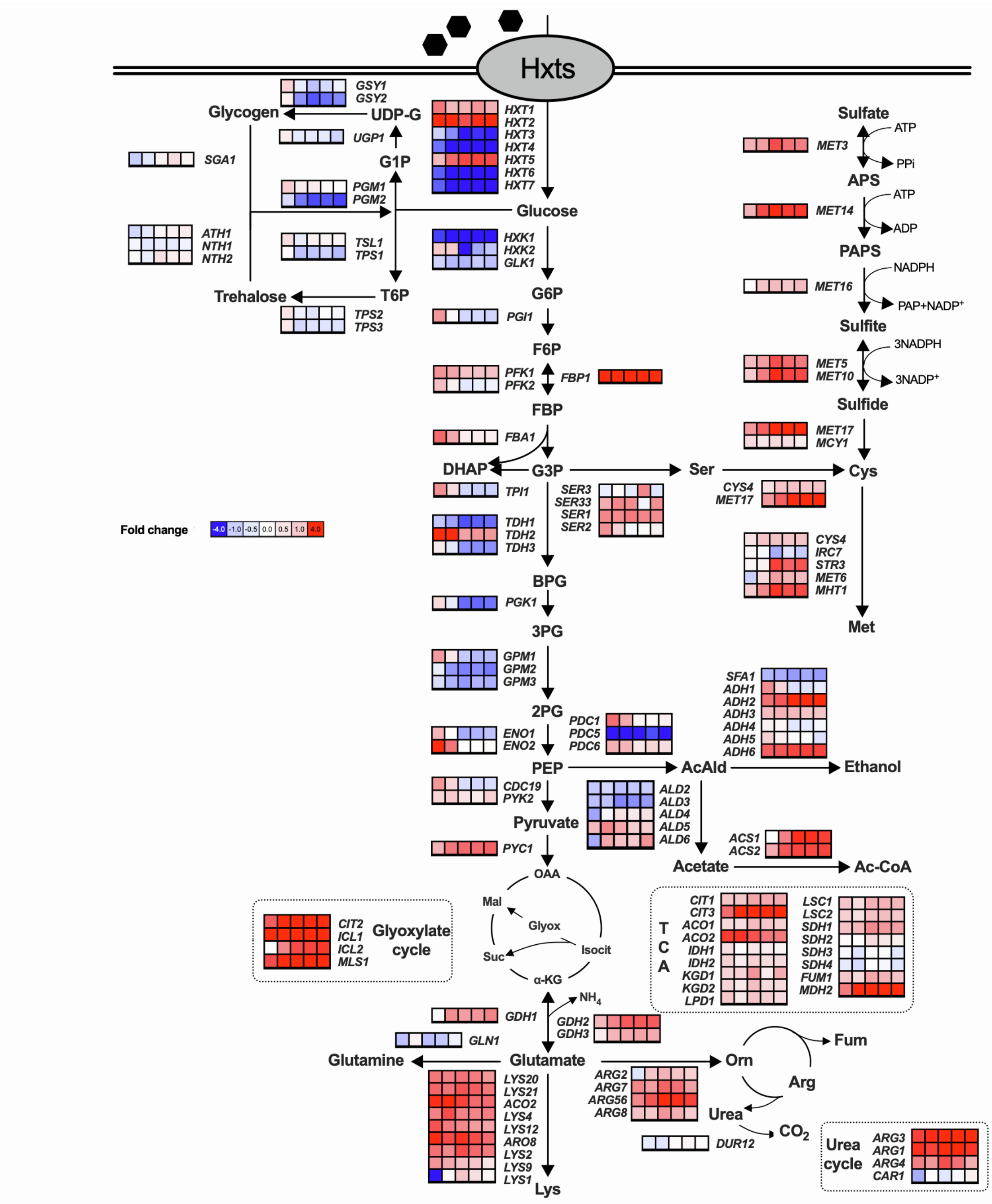

Fig. S9 Compared to FCF, the difference in gene expressions of BIF related to carbon metabolism (Glycolysis, Glycogen biosynthesis, Trehalose biosynthesis, Pyruvate metabolism, TCA, Glyoxylate cycle) and nitrogen metabolism (Cysteine and methionine biosynthesis, Glutamate biosynthesis, Lysine biosynthesis, and arginine biosynthesis). The sample names from left to right are BP4 vs FP4, B4I1 vs FP4, B4I2 vs FP4, B4I3 vs FP4, and B4I4 vs FP4. Abbreviations: HXTs, hexose transporters; G6P, Glucose-6-phosphate; F6P, Fructose-6-phosphate; FBP, Fructose-1,6-bisphosphate; G3P, Glyceraldehyde 3-phosphate; BPG, 1,3-bisphosphoglycerate; 3PG, 3-

phosphoglycerate; 2PG, 2-phosphoglycerate; PEP, Phosphoenolpyruvate; DHAP, Dihydroxyacetone phosphate; Ser, Serine; Cys, Cysteine; Met, Methionine; APS, Adenylyl sulfate; PAPS, 3-phosphoadenylyl sulfate; AcAld, Acetaldehyde; Ac-CoA, Acetyl-CoA; OAA, oxaloacetate; Iso, Isocitrate; α-KG, α-Ketoglutarate; Suc, Succinate; Mal, Malate; Lys, Lysine; Orn, Ornithine; Arg, Arginine; Fum, Fumarate.
